# VEGF-B ablation in pancreatic β-cells upregulates insulin expression without affecting glucose homeostasis or islet lipid uptake

**DOI:** 10.1038/s41598-020-57599-2

**Published:** 2020-01-22

**Authors:** Frank Chenfei Ning, Nina Jensen, Jiarui Mi, William Lindström, Mirela Balan, Lars Muhl, Ulf Eriksson, Ingrid Nilsson, Daniel Nyqvist

**Affiliations:** Division of Vascular Biology, Department of Medical Biochemistry and Biophysics, Karolinska Institutet, Stockholm, 171 65 Sweden

**Keywords:** Lipids, Cell signalling, Diabetes

## Abstract

Type 2 diabetes mellitus (T2DM) affects millions of people and is linked with obesity and lipid accumulation in peripheral tissues. Increased lipid handling and lipotoxicity in insulin producing β-cells may contribute to β-cell dysfunction in T2DM. The vascular endothelial growth factor (VEGF)-B regulates uptake and transcytosis of long-chain fatty acids over the endothelium to tissues such as heart and skeletal muscle. Systemic inhibition of VEGF-B signaling prevents tissue lipid accumulation, improves insulin sensitivity and glucose tolerance, as well as reduces pancreatic islet triglyceride content, under T2DM conditions. To date, the role of local VEGF-B signaling in pancreatic islet physiology and in the regulation of fatty acid trans-endothelial transport in pancreatic islet is unknown. To address these questions, we have generated a mouse strain where VEGF-B is selectively depleted in β-cells, and assessed glucose homeostasis, β-cell function and islet lipid content under both normal and high-fat diet feeding conditions. We found that *Vegfb* was ubiquitously expressed throughout the pancreas, and that β-cell *Vegfb* deletion resulted in increased insulin gene expression. However, glucose homeostasis and islet lipid uptake remained unaffected by β-cell VEGF-B deficiency.

## Introduction

Obesity is a major risk factor for development of insulin resistance, type 2 diabetes mellitus (T2DM) and other related metabolic complications. The adipose tissue functions as the major storage site for lipids under healthy conditions. In the case of obesity, excessive influx of unoxidized long-chain fatty acids (LCFA) exceeds the storage capacity of adipose tissue, leading to lipid spillover and accumulation in non-adipose tissues, such as the heart, skeletal muscle and pancreatic islets^1^. Ectopic lipid deposition in non-adipose tissue can have detrimental toxic effects at the cellular level and cause cellular dysfunction and destruction, a phenomenon termed as lipotoxicity^1^.

Pancreatic β-cells play a central role in glucose homeostasis by secreting insulin. Insulin release from β-cells is regulated by a sophisticated interplay between nutrients, hormones and neurotransmitters^2^. Development of T2DM is characterized by β-cell dysfunction and the inability to produce and secrete sufficient amounts of insulin to compensate for the increased need due to insulin resistance. Multiple mechanisms have been identified to contribute to β-cell failure, including mitochondrial dysfunction, ER stress, secretory dysfunction, dysfunctional lipid handling and glucolipotoxicity^3,4^. Free fatty acids (FFAs) have been shown to have both positive and negative effects on β-cell function depending on the concentration, duration, chain length and the degree of saturation. In obesity, elevated levels of FFAs caused impaired insulin secretion via increased oxidative stress and inflammation^5^, which may lead to progression and development of T2DM. Multiple *ex vivo* studies have accordingly demonstrated that prolonged exposure of pancreatic islets to FFAs resulted in decreased islet insulin content and impaired insulin gene expression^6–8^.

The vascular endothelial growth factor (VEGF)-B has been demonstrated to regulate LCFA trans-endothelial transport in a paracrine fashion particularly in tissues with high metabolic activity^9,10^. In adult mice, VEGF-B is highly expressed in heart and skeletal muscle but is also expressed at a lower level in other tissues, including pancreatic islets^9,11,12^. VEGF-B produced by the tissue cells acts on its receptors, neuropilin-1 (NRP-1) and VEGF receptor (VEGFR)-1, located on the endothelial cell surface, to increase the expression of the fatty acid transport proteins (FATP)-3 and FATP4, and subsequently facilitating endothelial cell LCFA uptake and transport into the surrounding tissue^9^. Further studies have also shown that targeting VEGF-B, by genetic deletion or by pharmacological inhibition, reduced tissue lipid accumulation, prevented dyslipidemia and improved insulin resistance^13,14^. Notably, total genetic ablation of VEGF-B (*Vegfb*^−/−^) also resulted in reduced pancreatic islet triglyceride accumulation and preserved β-cell mass in the *db/db* mouse model of T2DM^13^. However, the role of paracrine VEGF-B signaling in pancreatic islet physiology and pathology remains unexplored. In particular, the impact of β-cell derived VEGF-B on islet endothelial LCFA transport and uptake, is yet to be determined.

To address these questions, we have generated and characterized a novel mouse model where *Vegfb* is selectively depleted in pancreatic β-cells using the Cre/LoxP system. We examined the *Vegfb* expression pattern in the pancreas and found *Vegfb* to be ubiquitously expressed both in the endocrine and in the exocrine parts of the pancreas. Genetic depletion of VEGF-B selectively in β-cells decreased the overall islet *Vegfb* expression by 80%. Our data shows that mice with β-cell specific VEGF-B deficiency upregulates *Ins2* gene expression, whereas glucose homeostasis and islet lipid uptake were unaffected under conditions of both chow and high fat diet (HFD) feeding.

## Results

### VEGF-B is expressed in pancreatic islets and is significantly reduced in *Vegfb*^fl/fl^/RIP-Cre^+/−^ mice

**Figure 1:**
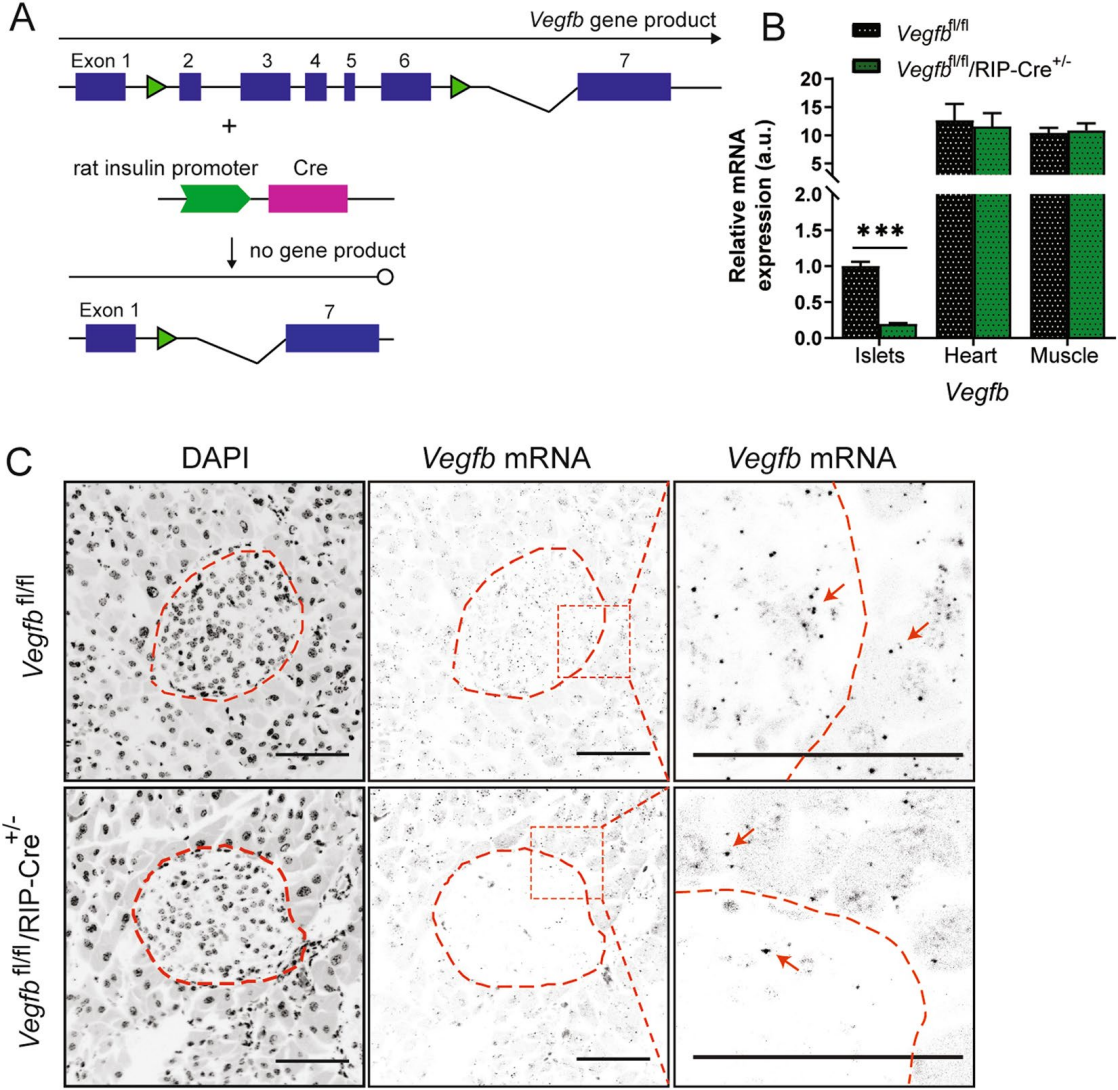
VEGF-B is expressed in pancreatic islets and is significantly reduced in *Vegfb*^fl/fl^/RIP-Cre^+/−^ mice. (**A**) Schematic diagram of Cre/LoxP based *Vegfb* selective deletion in β-cells. (**B**) Relative *Vegfb* mRNA levels in isolated pancreatic islets, heart tissue and skeletal muscle tissue from *Vegfb*^fl/fl^ and *Vegfb*^fl/fl^/RIP-Cre^+/−^ mice (n = 6). (**C**) Representative images of *Vegfb* mRNA transcripts (arrow) and nuclei (DAPI/black) in pancreas sections using RNAscope technology in *Vegfb*^fl/fl^ (n = 6) and *Vegfb*^fl/fl^/RIP-Cre^+/−^ (n = 6) mouse. Scale bars indicate 100 µm. Statistics: Mann-Whitney test (B). Data are presented as mean ± S.E.M; ***p < 0.001.

To investigate the role of VEGF-B signaling in pancreatic islet and β-cell function we generated β-cell specific VEGF-B deficient mice (*Vegfb*^fl/fl^/RIP-Cre^+/−^). *Vegfb*^fl/fl^/RIP-Cre^+/−^ mice were obtained by first creating a *Vegfb* floxed mouse line (*Vegfb*^fl/fl^) with two loxP sites inserted into the non-coding regions of the *Vegfb* gene, between exons 1 and 2, and exons 6 and 7, respectively (Fig. 1A). *Vegfb*^fl/fl^ mice were thereafter crossed with transgenic mice expressing the Cre recombinase under the rat insulin II promoter (RIP-Cre)^15^, where Cre recombination resulted in deletion of the floxed sequence and termination of *Vegfb* expression (Fig. 1A). To verify the deletion of *Vegfb* in β-cells of *Vegfb*^fl/fl^/RIP-Cre^+/−^ mice, total RNA was extracted from isolated pancreatic islets. Real-time quantitative PCR displayed an 80% reduction of *Vegfb* mRNA in *Vegfb*^fl/fl^/RIP-Cre^+/−^ islets compared to islets from *Vegfb*^fl/fl^ mice (Fig. 1B), indicating efficient recombination and reduction of *Vegfb* mRNA in β-cells. The expression levels of *Vegfb* in heart and skeletal muscle were around 10-fold higher compared to pancreatic islets, and unaffected by RIP-driven Cre expression (Fig. 1B).

To further investigate the expression and the localization of *Vegfb* mRNA in the pancreas, we performed RNAscope *in situ* hybridization on pancreatic sections from *Vegfb*^fl/fl^ and *Vegfb*^fl/fl^/RIP-Cre^+/−^ mice. Interestingly, *Vegfb* transcripts were detected at a similar abundance in cells of both the endocrine and exocrine parts of the pancreas in *Vegfb*^fl/fl^ mice (Fig. 1C). In sections from *Vegfb*^fl/fl^/RIP-Cre^+/−^ mice, the number of *Vegfb* transcripts was substantially reduced within the pancreatic islets as compared to *Vegfb*^fl/fl^ mice, whereas the number of transcripts in the surrounding exocrine tissue appeared similar in both groups (Fig. 1C). Taken together, these data demonstrate that *Vegfb* is expressed in both the endocrine and exocrine parts of the pancreas, and that Cre-induced recombination resulted in 80% reduction of the total islet *Vegfb* expression level in *Vegfb*^fl/fl^/RIP-Cre^+/−^ mice.

### β-cell specific *Vegfb* deletion results in increased insulin gene expression

**Figure 2:**
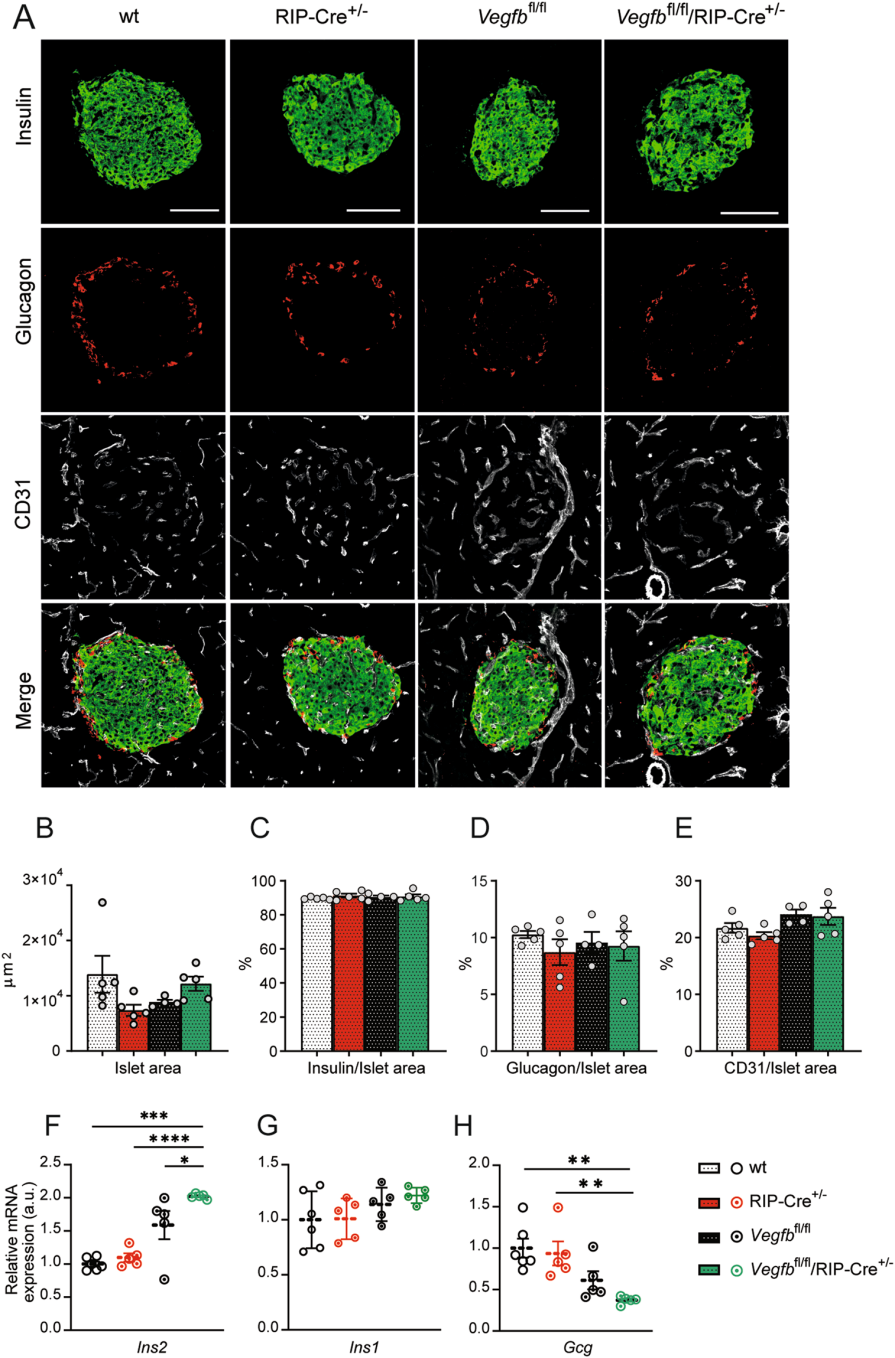
β-cell specific *Vegfb* deletion results in increased insulin expression. (**A**) Representative images of pancreatic islets stained for insulin, glucagon and CD31 from wt, RIP-Cre^+/−^, *Vegfb*^fl/fl^ and *Vegfb*^fl/fl^/RIP-Cre^+/−^ mice, scale bars indicate 100 µm (**B–E**) Quantification of islet area (μm^2^), insulin/islet area (%), glucagon/islet area (%) and CD31/islet area (%) from pancreatic sections of wt, RIP-Cre^+/−^, *Vegfb*^fl/fl^ and *Vegfb*^fl/fl^/RIP-Cre^+/−^ mice (n = 4–5 per group). (**F–H**) Relative *Ins2, Ins1, Gcg* mRNA levels in isolated islets from wt, RIP-Cre^+/−^, *Vegfb*^fl/fl^, and *Vegfb*^fl/fl^/RIP-Cre^+/−^ mice (n = 5–6 per group). Statistics: One-way ANOVA with Dunnett’s multiple comparison against *Vegfb*^fl/fl^/RIP-Cre^+/−^ group (B-H). Data are presented as mean ± S.E.M. *p < 0.05, **p < 0.01, ***p < 0.001, ****p < 0.0001.

To investigate the role of VEGF-B in islet physiology, we first examined islet morphology in mice with β-cell *Vegfb* deficiency by immunofluorescence staining of pancreatic sections. The RIP-Cre transgenic mouse model has been recognized to exhibit an altered metabolic phenotype when compared to wildtype (wt) mice, e.g. impaired glucose tolerance^16,17^. Therefore, in order to distinguish the effects caused by β-cell *Vegfb* deficiency from any effects derived from the RIP-Cre line, wt (Cre^−/−^), RIP-Cre^+/−^ and *Vegfb*^fl/fl^ mice were all used as controls throughout this study. Genetic ablation of *Vegfb* in β-cells did not affect pancreatic islet size as *Vegfb*^fl/fl^/RIP-Cre^+/−^ islets were similar to controls (Fig. 2A,B). Additionally, *Vegfb*^fl/fl^/RIP-Cre^+/−^ islets displayed a characteristic morphology with a core consisting of β-cells surrounded by peripherally located α-cells (Fig. 2A). Furthermore, we found no difference in the relative insulin and glucagon areas in *Vegfb*^fl/fl^/RIP-Cre^+/−^ islets, and the islet vessel area assessed by CD31 staining was also not affected by β-cell *Vegfb* deficiency (Fig. 2C–E).

To further elucidate the role of β-cell derived VEGF-B in islet physiology, we investigated the expression of genes related to glucose homeostasis. Interestingly, *Vegfb*^fl/fl^/RIP-Cre^+/−^ islets showed an increased level of *Ins2* mRNA expression in comparison to the other groups (Fig. 2E). *Ins1* mRNA expression was not affected (Fig. 2F). The level of glucagon (*Gcg)* mRNA expression was significantly reduced in *Vegfb*^fl/fl^/RIP-Cre^+/−^ islets when compared to wt and RIP-Cre^+/−^ islets, but not to *Vegfb*^fl/fl^ islets (Fig. 2G). Taken together, these results indicate that β-cell specific deletion of *Vegfb* results in increased insulin gene expression.

### β-cell specific *Vegfb* deficiency increases insulin secretion under low glucose conditions without affecting glucose homeostasis

**Figure 3:**
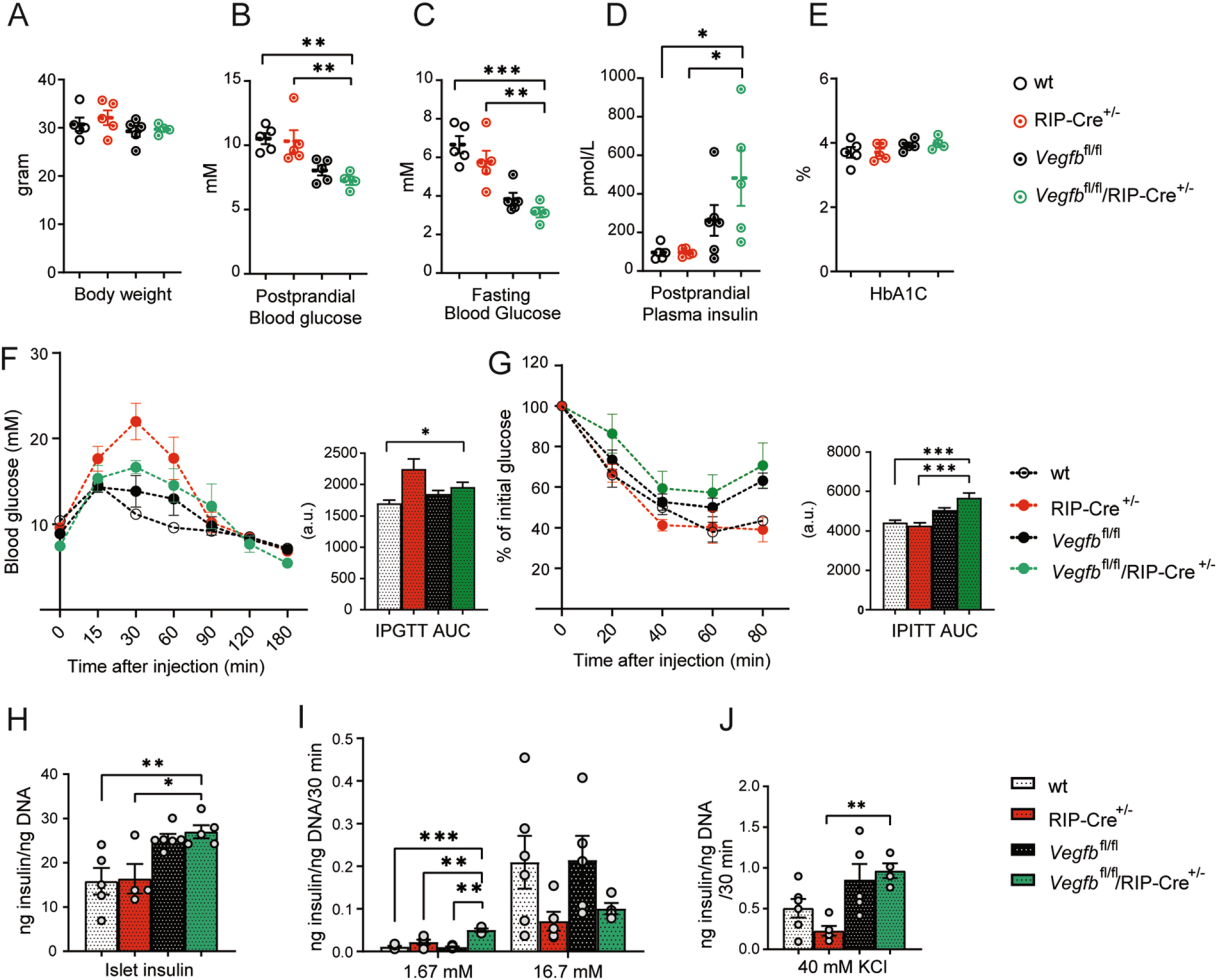
β-cell specific *Vegfb* deficiency increases insulin secretion under low glucose conditions without affecting glucose homeostasis. (**A**) Body weight measurement (**B**) Postprandial blood glucose levels (**C**) 12hrs fasting blood glucose levels (**D**) Postprandial plasma insulin levels and (**E**) HbA1c levels for wt, RIP-Cre^+/−^, *Vegfb*^fl/fl^, and *Vegfb*^fl/fl^/RIP-Cre^+/−^ mice (n = 5–6 per group). (**F**) Intraperitoneal glucose tolerance test and (**G**) Intraperitoneal insulin tolerance test with area under curve (AUC) analysis of wt, RIP-Cre^+/−^, *Vegfb*^fl/fl^ and *Vegfb*^fl/fl^/RIP-Cre^+/−^ mice (n = 5–10 per group). (**H**) Islet insulin content (**I**) Glucose stimulated insulin secretion (**J**) 40 mM KCl stimulated insulin secretion from wt, RIP-Cre^+/−^, *Vegfb*^fl/fl^ and *Vegfb*^fl/fl^/RIP-Cre^+/−^ islets (n = 4–6 per group). (H-J) All data have been normalized with islet DNA content. Statistics: One-way ANOVA with Dunnett’s multiple comparison against *Vegfb*^fl/fl^/RIP-Cre^+/−^ group (**A**,**C**–**J**). All data are presented as mean ± S.E.M. *p < 0.05, **p < 0.01, ***p < 0.001.

To address the role of β-cell selective *Vegfb* ablation on systemic glucose homeostasis and β-cell function, we characterized adult mice on chow diet. *Vegfb*^fl/fl^/RIP-Cre^+/−^ mice were healthy, fertile and displayed similar body weight as controls (Fig. 3A). Consistent with the observed increase in *Ins2* gene expression, *Vegfb*^fl/fl^/RIP-Cre^+/−^ mice showed decreased blood glucose levels in both postprandial and fasted state in comparison with both wt and RIP-Cre^+/−^ mice, though no significant difference was observed when compared to *Vegfb*^fl/fl^ mice (Fig. 3B,C). Consistent with this observation, the postprandial plasma insulin level was significantly higher in *Vegfb*^fl/fl^/RIP-Cre^+/−^ mice when compared to wt and RIP-Cre^+/−^ mice, but not in comparison to *Vegfb*^fl/fl^ mice (Fig. 3D). In contrast, measurements of glycated hemoglobin (HbA1c) indicated similar average blood glucose levels in all four groups (Fig. 3E). To further analyze glucose homeostasis in β-cell specific *Vegfb* deficient mice, we performed intra-peritoneal glucose and insulin tolerance tests (IPGTT and IPITT). *Vegfb*^fl/fl^/RIP-Cre^+/−^ mice displayed similar glucose tolerance as wt and *Vegfb*^fl/fl^ mice (Fig. 3F). Notably, RIP-Cre^+/−^ mice demonstrated impaired glucose clearance compared to wt mice, consistent with previous observations (Fig. 3F)^16,17^. The insulin tolerance test showed decreased insulin sensitivity in *Vegfb*^fl/fl^/RIP-Cre^+/−^ mice compared to wt and RIP-Cre^+/−^ mice, but not to *Vegfb*^fl/fl^ mice (Fig. 3G).

To further study the role of β-cell derived VEGF-B, insulin content was measured in isolated pancreatic islets. The insulin content was higher in *Vegfb*^fl/fl^/RIP-Cre^+/−^ islets when compared to wt and RIP-Cre^+/−^ islets, but not when compared with *Vegfb*^fl/fl^ islets (Fig. 3H). Next, we analyzed glucose-stimulated-insulin-secretion (GSIS) in isolated islets. Notably, under low glucose conditions, *Vegfb*^fl/fl^/RIP-Cre^+/−^ islets showed increased insulin secretion compared to the control groups (Fig. 3I). All groups responded with increased insulin secretion in response to high glucose stimulation, while no significant difference was found between the groups (Fig. 3I). Following GSIS, β-cells were depolarized by KCl which resulted in a higher level of insulin secretion from *Vegfb*^fl/fl^/RIP-Cre^+/−^ islets compared to RIP-Cre^+/−^ islets, but similar compared to *Vegfb*^fl/fl^ islets (Fig. 3J). Collectively, these results suggest that β-cell specific deletion of *Vegfb* increase insulin secretion under low glucose conditions but does not affect glucose tolerance or insulin sensitivity in mice kept on chow diet.

### Vegfb depletion in β-cells does not affect insulin gene expression under HFD conditions

**Figure 4:**
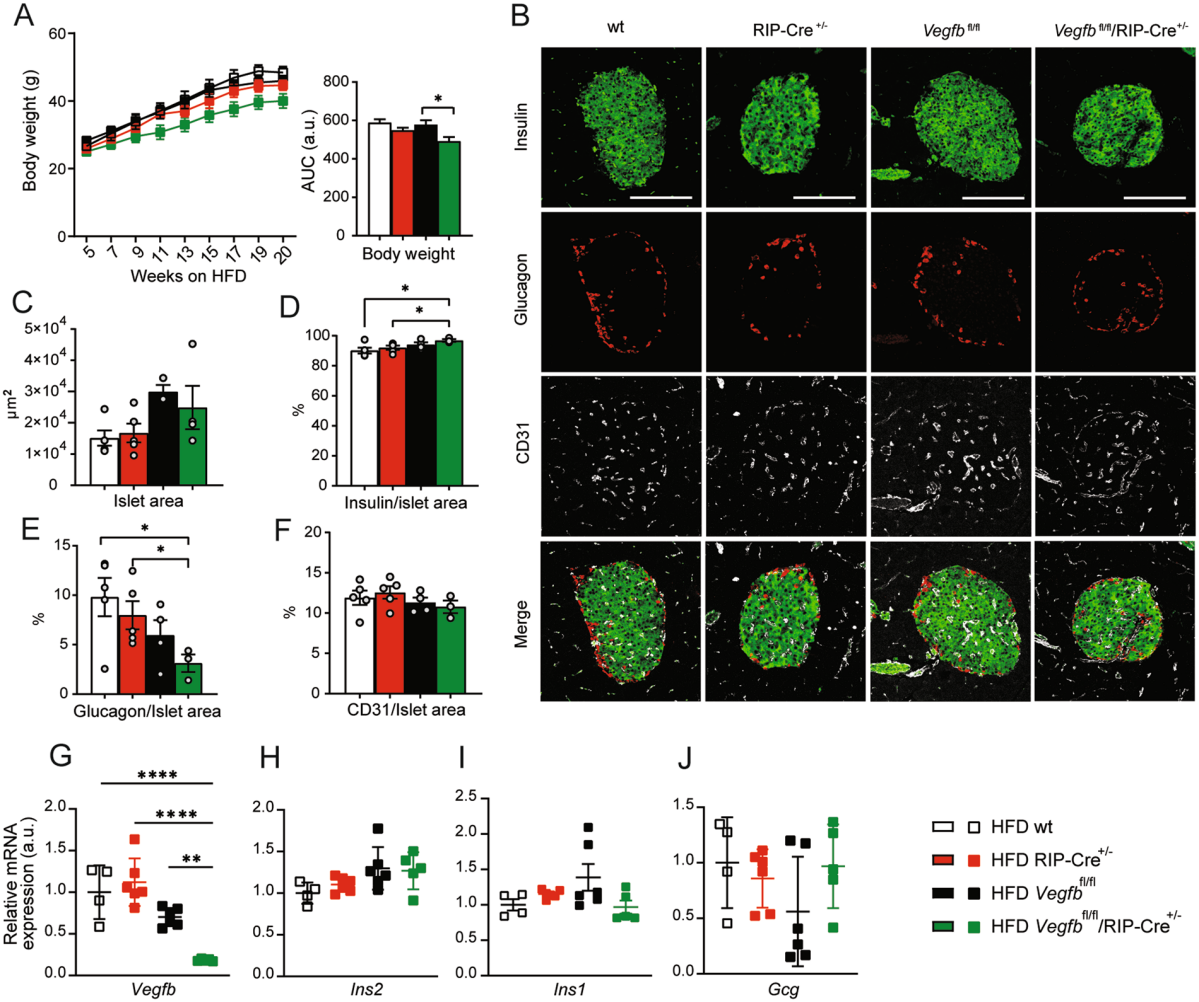
*Vegfb* depletion in β-cells does not affect insulin gene expression under HFD conditions. (**A**) Bi-weekly measurement of body weight and AUC analysis of HFD wt, RIP-Cre^+/−^, *Vegfb*^fl/fl^ and *Vegfb*^fl/fl^/RIP-Cre^+/−^ mice (n = 4–12 per group). (**B**) Representative images of pancreatic islets stained with insulin, glucagon and CD31 from HFD wt, RIP-Cre^+/−^ *Vegfb*^fl/fl^ and *Vegfb*^fl/fl^/RIP-Cre^+/−^ mice. Scale bars indicate 100 µm. (**C–F**) Quantification of islet area (μm^2^), insulin/islet area (%), glucagon/islet area (%) and CD31/islet area (%) from pancreatic sections of HFD wt, RIP-Cre^+/−^, *Vegfb*^fl/fl^ and *Vegfb*^fl/fl^/RIP-Cre^+/−^ mice (n = 4–5 per group). (**G–J**) Relative *Vegfb, Ins2, Ins1, Gcg* mRNA level in isolated islets from HFD wt, RIP-Cre^+/−^, *Vegfb*^fl/fl^ and *Vegfb*^fl/fl^/RIP-Cre^+/−^ mice (n = 4–6 per group). Statistics: One-way ANOVA with Dunnett’s multiple comparison against *Vegfb*^fl/fl^/RIP-Cre^+/−^ group (A,C–J). All data are presented as mean ± S.E.M. *p < 0.05, **p < 0.01, ****p < 0.0001.

Previous investigations have demonstrated that genetic knockout of *Vegfb* (*Vegfb*^−/−^*)*, or pharmacological inhibition of VEGF-B, resulted in restoration of insulin sensitivity and glucose tolerance, as well as preservation of insulin production and pancreatic islet morphology in the *db/db* T2DM mouse model^13^. To address whether selective β-cell *Vegfb* deficiency could recapitulate the protective effects observed on insulin production and islet morphology obtained by systemic VEGF-B inhibition under obese conditions, we subjected mice to high-fat diet (HFD) feeding for 20 weeks, starting from 5 weeks of age. All groups responded with increased body weight when subjected to HFD (Fig. 4A). Interestingly, HFD *Vegfb*^fl/fl^/RIP-Cre^+/−^ mice demonstrated a reduced weight gain compared to HFD *Vegfb*^fl/fl^ mice, but indifferent from RIP-Cre controls (Fig. 4A). Next, we evaluated islet size and morphology by immunofluorescence staining of pancreatic sections. Quantification of the islet area showed no differences among the different groups on HFD (Fig. 4B,C). In addition, *Vegfb*^fl/fl^/RIP-Cre^+/−^ mice subjected to HFD displayed increased relative insulin area and decreased relative glucagon area when compared to wt and RIP-Cre^+/−^ mice (Fig. 4D,E). However, HFD *Vegfb*^fl/fl^/RIP-Cre^+/−^ islets did not differ from HFD *Vegfb*^fl/fl^ islets in terms of the relative insulin and glucagon areas (Fig. 4D,E), and the islet vessel area was also similar in *Vegfb*^fl/fl^/RIP-Cre^+/−^ islets when compared to the controls (Fig. 4F).

Next, we analyzed the effect of β-cell *Vegfb* deficiency on islet gene expression in response to HFD. *Vegfb*^fl/fl^/RIP-Cre^+/−^ islets retained a similar reduction of *Vegfb* expression on HFD, as observed on chow diet (Fig. 4G). In contrast to mice on chow diet, *Ins2* expression was indifferent from the control groups (Fig. 4H). *Ins1* mRNA expression was also unaffected under HFD conditions (Fig. 4I). *Gcg* expression also remained unchanged in islets from *Vegfb*^fl/fl^/RIP-Cre^+/−^ mice on HFD compared to controls (Fig. 4J). In summary, these data demonstrate that β-cell *Vegfb* deficiency did not affect pancreatic islet morphology or insulin gene expression under HFD condition.

### β-cell specific *Vegfb* deficiency does not affect glucose homeostasis under HFD conditions

**Figure 5:**
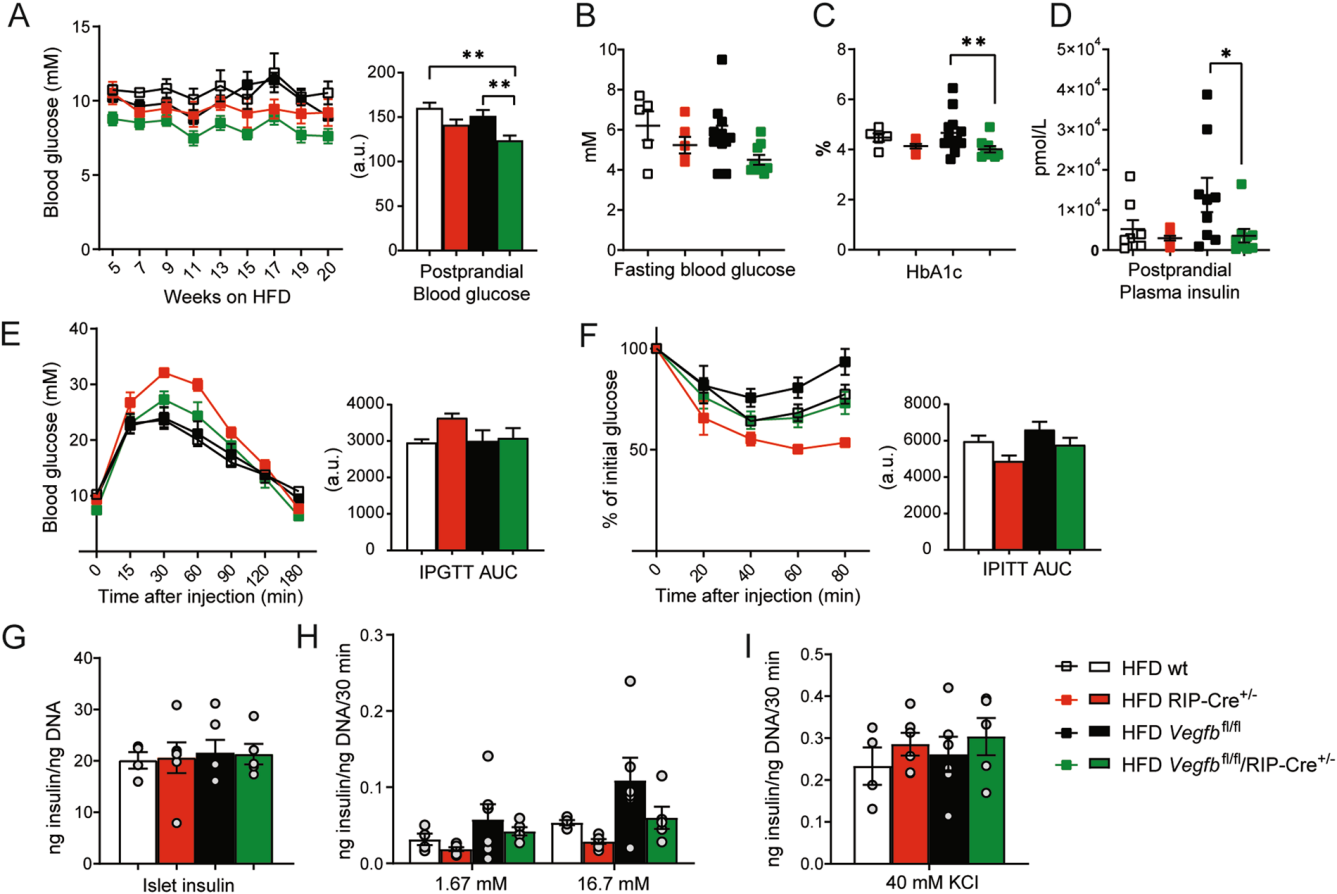
β-cell specific *Vegfb* deficiency does not affect glucose homeostasis under HFD conditions. (**A**) Bi-weekly measurement of postprandial blood glucose and AUC analysis of HFD wt, RIP-Cre^+/−^, *Vegfb*^fl/fl^ and *Vegfb*^fl/fl^/RIP-Cre^+/−^ mice (n = 4–12 per group). (**B–D**). Fasting blood glucose, HbA1c, and postprandial plasma insulin level of HFD wt, RIP-Cre^+/−^, *Vegfb*^fl/fl^ and *Vegfb*^fl/fl^/RIP-Cre^+/−^ mice (n = 5–12 per group). (**E,F**) Intraperitoneal glucose and insulin tolerance tests and area under curve analysis of HFD wt, RIP-Cre^+/−^, *Vegfb*^fl/fl^ and *Vegfb*^fl/fl^/RIP-Cre^+/−^ mice (n = 4–12 per group). (**G**) Isolated islet insulin content from HFD wt, RIP-Cre^+/−^, *Vegfb*^fl/fl^ and *Vegfb*^fl/fl^/RIP-Cre^+/−^ mice (n = 4–6 per group). Data normalized with islet DNA content. (**H**) Glucose stimulated insulin secretion and (**I**) 40 mM KCl stimulated insulin secretion from isolated islets from HFD wt, RIP-Cre^+/−^, *Vegfb*^fl/fl^ and *Vegfb*^fl/fl^/RIP-Cre^+/−^ mice (n = 4–6 mice/group). Secreted insulin normalized with islets DNA content. Statistics: One-way ANOVA with Dunnett’s multiple comparison against *Vegfb*^fl/fl^/RIP-Cre^+/−^ group (A–I). All data are presented as mean ± S.E.M. *p < 0.05, **p < 0.01.

Next, we evaluated if β-cell specific *Vegfb* deficiency would affect glucose homeostasis under HFD conditions. Interestingly, monitoring of postprandial blood glucose levels indicated that *Vegfb*^fl/fl^/RIP-Cre^+/−^ mice maintained a lower blood glucose level as compared to wt and *Vegfb*^fl/fl^ controls, although not significantly different from RIP-Cre^+/−^ mice (Fig. 5A). To further investigate glucose homeostasis, we measured fasting blood glucose and HbA1c, and *Vegfb*^fl/fl^/RIP-Cre^+/−^ mice showed lower but not significantly different levels of fasting blood glucose when compared to the rest of the groups (Fig. 5B). HbA1c was lower in *Vegfb*^fl/fl^/RIP-Cre^+/−^ mice when compared to *Vegfb*^fl/fl^ mice, but not when compared to wt and RIP-Cre^+/−^ mice (Fig. 5C). Measurements of both postprandial and fasting plasma insulin levels were lower in HFD *Vegfb*^fl/fl^/RIP-Cre^+/−^ mice in comparison with *Vegfb*^fl/fl^ mice, but similar to the other groups (Fig. 5D and Supplemental Fig. S1A). Moreover, *Vegfb*^fl/fl^/RIP-Cre^+/−^ mice showed similar performance in both IPGTT and IPITT as the other groups (Fig. 5E,F). Of note, RIP-Cre^+/−^ mice displayed impaired glucose tolerance also under HFD conditions (Fig. 5E).

We further investigated the impact of β-cell *Vegfb* depletion on insulin content and GSIS in isolated islets following 20 weeks of HFD. Measurements showed similar insulin content (Fig. 5G) and comparable GSIS (Fig. 5H) among all groups. Likewise, β-cell depolarization with KCl resulted in a comparable release (Fig. 5I). In summary, these data suggest that β-cell *Vegfb* ablation does not affect overall glucose homeostasis in mice subjected to HFD.

### Pancreatic islet VEGF-B expression is not affected by increased metabolic activity and does not affect islet FATP3 and FATP4 expression or lipid uptake

**Figure 6:**
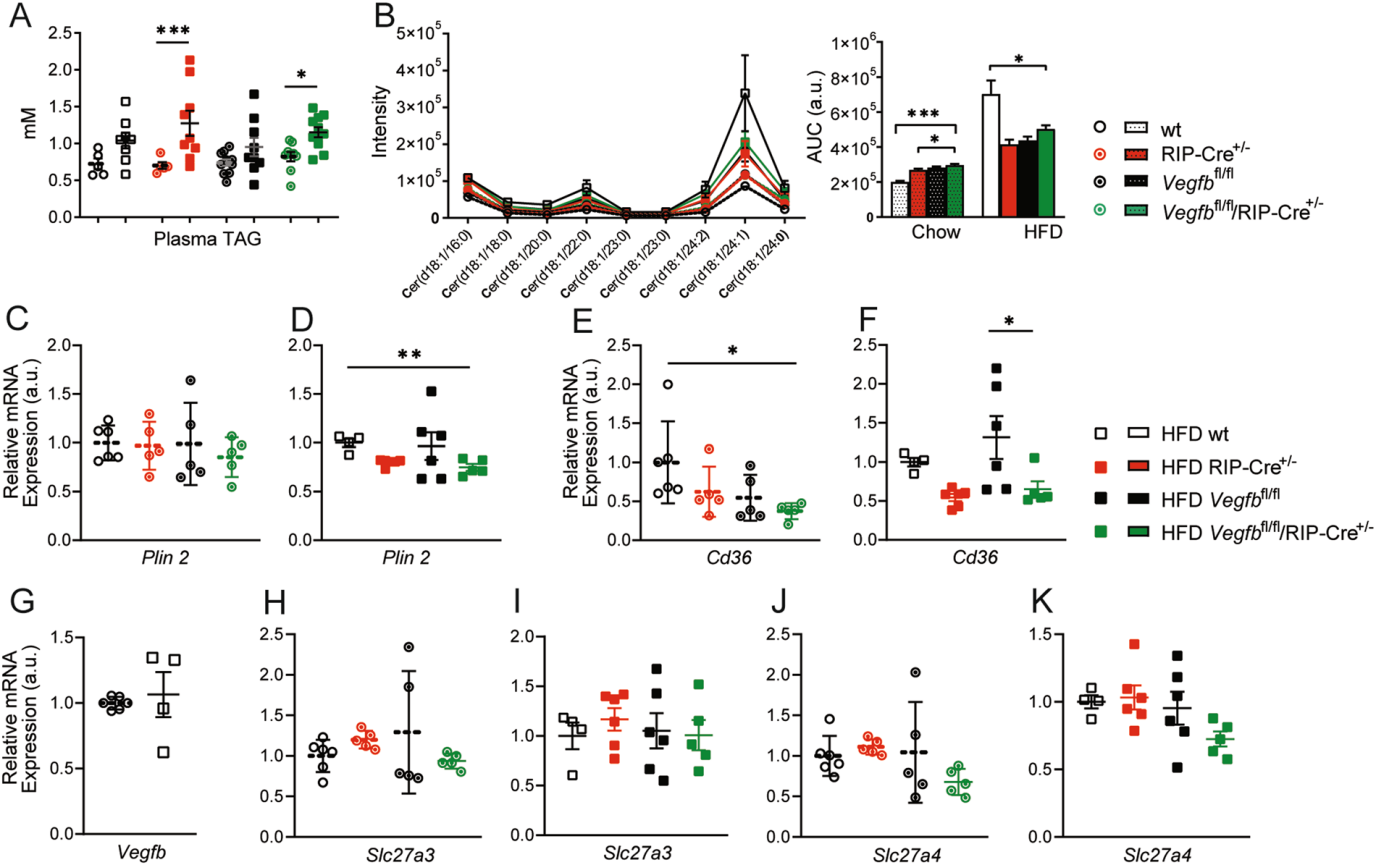
Pancreatic islet VEGF-B expression is not affected by increased metabolic activity and does not affect FATP3 and FATP4 expression or islet lipid uptake. (**A**) Comparison of plasma triglyceride level in chow vs. HFD fed wt, RIP-Cre^+/−^, *Vegfb*^fl/fl^ and *Vegfb*^fl/fl^/RIP-Cre^+/−^ mice (n = 5–11 per group). (**B**) UFLC-MS analysis of islet ceramide species and area under curve (AUC) analysis of islet ceramide content from chow and HFD fed wt, RIP-Cre^+/−^, *Vegfb*^fl/fl^ and *Vegfb*^fl/fl^/RIP-Cre^+/−^ mice (n = 4–6 per group). (**C,D**) Relative *Plin2* mRNA levels in isolated islets from chow and HFD fed wt, RIP-Cre^+/−^, *Vegfb*^fl/fl^ and *Vegfb*^fl/fl^/RIP-Cre^+/−^ mice (n = 4–6 per group). (**E,F**) Relative *Cd36* mRNA levels in isolated islets from chow and HFD fed wt, RIP-Cre^+/−^, *Vegfb*^fl/fl^ and *Vegfb*^fl/fl^/RIP-Cre^+/−^ mice (n = 4–6 per group). (**G**) Comparison of relative *Vegfb* mRNA levels in islets from chow vs. HFD fed wt mice (n = 4–6 per group). (**H,I**) Relative *Slc27a3* mRNA levels in isolated islets from chow and HFD fed wt, RIP-Cre^+/−^, *Vegfb*^fl/fl^ and *Vegfb*^fl/fl^/RIP-Cre^+/−^ mice (n = 4–6 per group). (**J,K**) Relative *Slc27a4* mRNA levels in isolated islets from chow and HFD fed wt, RIP-Cre^+/−^, *Vegfb*^fl/fl^ and *Vegfb*^fl/fl^/RIP-Cre^+/−^ mice (n = 4–6 per group). Statistics: Mann-Whitney test (A,G), one-way ANOVA with Dunnett’s multiple comparison against *Vegfb*^fl/fl^/RIP-Cre^+/−^ group (B-F, H-K). All data are presented as mean ± S.E.M. *p < 0.05, **p < 0.01, ***p < 0.001.

Paracrine VEGF-B signaling has been demonstrated to mediate endothelial LCFA uptake via regulation of FATP3 and FATP4 expression in heart, skeletal muscle and kidney glomeruli^9,13,14^. To address whether paracrine VEGF-B signaling modulates pancreatic islet FATP-3 and -4 expression and fatty acid uptake, we analyzed islet lipid content and indicators for islet fatty acid uptake. We first measured plasma TAG levels to characterize the systemic lipid load obtained by 20 weeks of HFD feeding. We found equal plasma TAG levels in the four groups on each diet condition (Fig. 6A). As expected, the plasma TAG levels increased in response to HFD in all groups, however, the increase was mild and only RIP-Cre^+/−^ and *Vegfb*^fl/fl^/RIP-Cre^+/−^ mice displayed significantly higher levels compared to when fed chow diet (Fig. 6A).

Upon entry into β-cells, FFAs either undergo β-oxidation, or they will be directed to enter the glycerolipid/FFA cycle to generate triacylglycerol (TAG) which promote lipid droplet (LD) formation or, alternatively, participate in sphingolipid synthesis to generate a number of different metabolites, such as ceramide^18^. To address whether VEGF-B mediated fatty acid transport machinery operate in pancreatic islets and particularly to what extent VEGF-B is involved in β-cell fatty acid uptake, we measured islet ceramide content and *Plin2* expression (a marker for LD formation) as indicators for islet fatty acid uptake. A series of ceramide species in isolated islets were measured by LC-MS. We found that the ceramide content was increased in islets from all groups following 20 weeks of HFD in comparison to islets from mice kept on chow diet (Fig. 6B and Supplementary Fig. S1B), reflecting the increased systemic lipid load acquired by HFD feeding. However, we could not determine any sign of decreased ceramide formation in *Vegfb*^fl/fl^/RIP-Cre^+/−^ islets compared with islets from the control groups in either diet condition (Fig. 6B), indicating that β-cell *Vegfb* depletion did not affect ceramide formation in pancreatic islets. In addition, we also observed no difference in *Plin2* expression among the groups on chow diet (Fig. 6C), and decreased expression of *Plin2* only in HFD *Vegfb*^fl/fl^/RIP-Cre^+/−^ islets when compared to HFD wt islet, indicating that islet LD content was unaffected by β-cell specific *Vegfb* deficiency (Fig. 6D).

FFA uptake into islet β-cells is suggested to involve the CD36 translocase^19^. To further understand the role of VEGF-B in islet fatty acid uptake and lipid handling, we examined the expression level of *Cd36* in isolated islets. In chow diet condition, islets from *Vegfb*^fl/fl^/RIP-Cre^+/−^ mice exhibited lower *Cd36* expression compared to wt mice, whereas the *Cd36* expression was unaffected compared to the other controls (Fig. 6E). In response to HFD feeding for 20 weeks, *Cd36* was upregulated in *Vegfb*^fl/fl^ islets, but similar in the other groups, suggesting a limited effect of *Vegfb* ablation on *Cd36* expression (Fig. 6F).

The expression of *Vegfb* has been linked to the metabolic activity of the tissue^9^, and found to be 4-fold up-regulated in muscle tissue in response to HFD when compared to chow diet^20^. To analyze the impact of increased metabolic activity on pancreatic islet *Vegfb* expression, we compared wt mice on chow diet versus HFD. Notably, islet *Vegfb* expression was not affected by exposure to HFD (Fig. 6G), suggesting that islet *Vegfb* expression is not coupled with increased β-cell metabolic activity in response to HFD challenge. To further explore the role of β-cell derived VEGF-B expression, we investigated the impact of β-cell *Vegfb* deletion on the expression of the VEGF-B target genes *Slc27a3* (FATP3) and *Slc27a4* (FATP4) in pancreatic islets. No effect could be observed on *Slc27a3* expression in pancreatic islets from *Vegfb*^fl/fl^/RIP-Cre^+/−^ mice subjected to chow or HFD conditions (Fig. 6H,I). The expression of *Slc27a4* was likewise not altered in *Vegfb*^fl/fl^/RIP-Cre^+/−^ islets when compared to control groups (Fig. 6J,K). Furthermore, no alteration due to β-cell *Vegfb* deletion could be observed on the expression levels of the endothelial VEGF-B receptors *Nrp1* and *Flt1*, and neither on the expression of *Vegfa, Flk1* or *Pecam1* (Supplementary Fig. S2). In summary, these data indicate that β-cell derived VEGF-B expression is not affected by increased metabolic activity and does not affect islet FATP3 and FATP4 expression or lipid uptake.

## Discussion

Herein we describe and characterize a novel mouse model where VEGF-B is selectively ablated in pancreatic β-cells. Our findings demonstrate that VEGF-B is ubiquitously expressed throughout the pancreas and reduced by 80% in pancreatic islets following deletion in β-cells. Interestingly, *Vegfb* ablation from β-cells resulted in upregulation of *Ins2* gene expression.

The biological function of VEGF-B has long been poorly understood. Despite its sequence homology with VEGF-A^21^, its role in angiogenesis is limited to conditions of over-expression^22–24^. The findings that regulation of VEGF-B expression is coupled with metabolic activities hinted towards a potential role of VEGF-B in energy metabolism^9,25,26^. Genetic *Vegfb* deletion (*Vegfb*^−/−^*)*, or pharmacological inhibition of VEGF-B signaling using neutralizing antibodies, in a T2DM mouse model (*db/db*)^13^ restored insulin sensitivity and improved glucose tolerance correlating with preserved islet architecture and improved β-cell function. These findings suggested that inhibition of VEGF-B signaling could potentially prevent and protect islets from diabetes-induced injury. However, the role of pancreatic islet-derived paracrine VEGF-B signaling in islet physiology remains unclear.

To study the role of VEGF-B signaling in pancreatic islets, we utilized the Cre/LoxP system to generate a mouse line with conditional deletion of *Vegfb* in pancreatic β-cells (*Vegfb*^fl/fl^/RIP-Cre^+/−^). The RIP-Cre driver mice have been widely used to study β-cell physiology due to high recombination efficiency in β-cells^27^. However, unexpected metabolic phenotypes associated with the RIP-Cre line have been discovered in later years, among which the most common one is glucose intolerance^16,28^, also reproduced in our present study. Recent investigations unveiled that the unpredicted expression of the human growth hormone (*h*GH) minigene, incorporated into the transgenic construct for the sole purpose of enhancing Cre expression, is responsible for the observed metabolic phenotypes^17^. As the RIP-Cre line was indispensable for achieving β-cell specific *Vegfb* deletion, we therefore applied the criteria that the effects observed in β-cell *Vegfb* deficient mice (*Vegfb*^fl/fl^/RIP-Cre^+/−^) had to differ from *Vegfb*^fl/fl^ controls as well as RIP-Cre^+/−^ and wt mice in order to accurately assess effects stemming from β-cell *Vegfb* deficiency. Hence, statistical validation was performed comparing *Vegfb*^fl/fl^/RIP-Cre^+/−^ mice towards all three control groups.

By analyzing isolated islet mRNA, we found *Vegfb* to be expressed in pancreatic islets, confirming previous studies^29^. In relation to other murine tissues, islet *Vegfb* content was found to be 10-fold lower than the level in cardiac and skeletal muscle. RIP-Cre driven recombination in *Vegfb*^fl/fl^ mice resulted in over 80% reduction in islet *Vegfb* expression, indicating efficient recombination and a major contribution of β-cell derived VEGF-B to the total islet pool of VEGF-B. Furthermore, *in situ* hybridization using RNAscope technology confirmed these results, and also demonstrated ubiquitous *Vegfb* expression in the neighboring exocrine pancreas. These observations are in line with mouse and human single cell transcriptome studies, where *Vegfb* has been found to be expressed in all pancreatic endocrine cells and acinar cells^30,31^.

Insulin expression has been shown to be transcriptionally regulated and tightly controlled by blood glucose^32^. In a synergistic manner, the Pdx-1, PAX6, NeuroD1 and MafA transcription factors regulate insulin gene expression in β-cells^33–35^. Unlike other mammalian species, insulin in mice and rats is encoded by a two-gene, *Ins1* and *Ins2*, system^36,37^. *Ins1* is a rodent specific retrogene and contains only one intron present in the *Ins2* gene^36^, while *Ins2* exhibits more structural and functional similarity to the insulin gene of other mammalian species^37^. Moreover, *Ins2* but not *Ins1* is required to maintain normal insulin secretion as the *Ins2* knockout mouse become insulin deficient and diabetic^38^. The finding that β-cell *Vegfb* deficiency upregulates islet *Ins2*, whereas *Ins1* gene expression remained unaffected, could indicate a potential VEGF-B dependent transcriptional activation of *Ins2*, However, as current knowledge about the role of VEGF-B in the regulation of insulin gene expression is lacking, hence further studies are warranted in order to elucidate this possibility.

Consistent with the gene expression data, *ex vivo* analysis of *Vegfb*^fl/fl^/RIP-Cre^+/−^ islets also displayed enhanced insulin release in low glucose conditions. However, high glucose stimulation revealed no difference in insulin secretion from β-cell *Vegfb* deficient islets. Furthermore, systemic glucose tolerance was not affected by β-cell *Vegfb* deficiency. These data suggest that pancreatic islet VEGF-B signaling may not contribute to insulin-regulated glucose homeostasis, and the increased insulin release under low glucose condition are likely due to a cell autonomous effect and possibly conferred by an upregulated *Ins2* gene expression.

HFD feeding of C57BL/6 J mice induces obesity, hyperinsulinemia and mildly elevated blood glucose levels, consistent with an increased metabolic challenge^39^. In line with these observations, HFD feeding in our study also resulted in significant weight gain and a mild increase in blood glucose in all groups. In contrast to chow diet conditions, the level of *Ins2* expression was not increased in HFD *Vegfb*^fl/fl^/RIP-Cre^+/−^ islets compared to control islets. The *Vegfb*^fl/fl^/RIP-Cre^+/−^ mice however displayed a tendency for lowered postprandial glucose levels during HFD, but showed no difference in islet insulin release during basal or stimulated conditions *ex vivo*. The lack of effect on insulin gene expression in HFD condition may be explained by functional compensation, such as increased islet mass and size or altered energy metabolism induced by HFD^39^ which in turn could affect insulin gene expression^39–41^. In addition, and in contrast to previous observation in muscle where *Vegfb* expression was upregulated in HFD^20^, pancreatic islet *Vegfb* levels did not change in response to HFD feeding, further suggesting lack of a direct link between metabolic activity and *Vegfb* expression in β-cells.

Obesity-induced dyslipidemia has been linked to increased β-cell fatty acid uptake, neutral lipid accumulation and lipotoxicity^42^. Systemic blocking of VEGF-B using neutralizing antibodies has been shown to reduce TAG accumulation in islets in *db/db* T2DM mice^13^. In our study, we examined islet ceramide content and *Plin2* gene expression to evaluate if β-cell *Vegfb* deficiency affected islet neutral lipid content. *Plin2* is a member of the perilipin family of LD surface proteins and its level has been demonstrated to correlate with cellular TAG storage^43^. HFD feeding resulted in elevated plasma triglyceride levels, although not reaching significance for all groups. Significantly increased ceramide levels were detected in the islets from all groups, further supporting enhanced exposure to circulating lipids in response to HFD feeding. However, β-cell *Vegfb* ablation did not affect the islet ceramide content, nor *Plin2* expression level. Additionally, expression of the fatty acid transporter *Cd36* was also unaffected by β-cell *Vegfb* deficiency. Moreover, we did not observe downregulation of the VEGF-B target genes encoding FATP3 and FATP4 in response to β-cell *Vegfb* ablation. Collectively, these data indicate that in contrast to responses in *e.g*. cardiac and skeletal muscle, paracrine VEGF-B signaling in pancreatic islets does not target islet fatty acid uptake and lipotoxicity. Furthermore, this suggests that the beneficial effects observed by global and systemic VEGF-B inhibition correlating with reduced lipid deposition in peripheral tissues leading to increased tissue glucose uptake and maintained normoglycemia are more likely explained by the accompanying reduction in plasma triglycerides; a phenotype not recapitulated in our *Vegfb*^fl/fl^/RIP-Cre^+/−^ mice^13^. Importantly, in support of a role of VEGF-B signaling in glucose homeostasis, a clinical study on T2DM patients found significantly higher plasma VEGF-B levels in patients with impaired glucose regulation versus patients with normal glucose tolerance^44^.

Contribution of VEGF-B from exocrine pancreatic tissue may also affect the islet phenotype, as alternative splicing of the *Vegfb* gene gives rise to two isoforms, among which VEGF-B_186_ is freely diffusible^45^. Furthermore, LoxP flanking of the *Vegfb* gene could also affect alternative splicing or protein translation, possibly affecting a number of different measurements. However, it was beyond the scope of the current study to characterize these aspects in full. Nonetheless this demonstrates the necessity to use appropriate controls when using this and other tissue specific knockouts in the future.

To our knowledge this is the first study to comprehensively characterize the effects of selective *Vegfb* deficiency in pancreatic β-cells. In conclusion we found that β-cell specific ablation of VEGF-B increases islet insulin expression and release under basal conditions. Additionally, our data indicates that local paracrine VEGF-B signaling in pancreatic islets, in contrast to a systemic VEGF-B targeting approach, shows limited therapeutic effect on overall systemic glucose homeostasis.

## Material and Methods

### Animals

*Vegfb* floxed mice (*Vegfb*^fl/fl^) on C57BL/6J background were generated by Taconic Artemis. To generate mice with *Vegfb* selective deletion in insulin producing β-cells, *Vegfb*^fl/fl^ mice have been intercrossed with a mouse line that express Cre under the rat insulin promoter *B6.Cg-Tg(Ins2-Cre)25Mgn/J*^15^. *Vegfb*^fl/fl^ mice were bred by mating with heterozygous RIP-Cre mice. Age-matched male mice were used throughout the study. Experimental endpoint for both chow and HFD fed mice were at 25 weeks of age.

Mice were fed with standard chow diet or a high-fat diet (HFD) containing 60% calories from fat (Research Diet, D12492). All mice had *ad libitum* access to food and water and were housed in standard cages with 12 hours light/dark cycles. All animal procedures were performed in accordance with relevant guidelines and regulations and approved by the Stockholm North Ethical Committee on Animal Research, Sweden.

### Blood glucose measurements and tolerance tests

Blood glucose measurement was performed as described before^13^. Briefly, postprandial blood glucose levels and body weight of mice were measured bi-weekly after the removal of food for 2 h. For fasting blood glucose, mice were starved for 12 h before measurement. Mice were starved for 3 hours prior to IPGTT and IPITT. 1 mg D-glucose per gram body weight, or 0.75 mU insulin per gram body weight were injected intraperitoneally (IP), where after blood samples were taken at indicated time-points. Blood glucose were measured from tail vein using a Bayer Contour glucose meter. Glycated hemoglobin was measured from tail vein blood using the HbA1c kit (Siemens) and analyzed by Siemens DCA Vantage Analyzer.

### Plasma analysis

Postprandial state mice were anaesthetized with isoflurane and blood was collected from the right atrium. For fasting state mice, blood was collected from the tail. Collected blood was centrifuged at 2000 g at 20 °C for 7 minutes. After centrifugation, the plasma fractions were recovered and frozen in aliquots at −80 °C. Plasma insulin was measured by Mercodia Mouse Insulin Elisa kit and plasma TAG level was determined using the Thermo Scientific Infinity Triglyceride kit according to manufacturer’s instructions.

### Glucose stimulated insulin secretion and islet insulin content

Pancreatic islets were isolated by collagenase digestion as previously described^46^, and further purified by hand picking. Islets were then either immediately frozen for measurement of insulin content, or further cultured in RPMI 1640 medium (Life technologies) supplemented with Penicillin Streptomycin (10,000 units/ml Penicillin, 10,000 µg/ml Streptomycin; Life technologies) and 10% (v/v) fetal bovine serum (Life technologies) in a designated cell culture incubator at 37 °C, 5% CO_2_ at ambient oxygen tension (20.9%). After 24 hours, 3 groups of 10 islets each were transferred into pre-gassed (95% O_2_, 5% CO_2_) Krebs-Ringer bicarbonate buffer supplemented with 10 mM HEPES (Sigma-Aldrich) and 0.2% BSA (Saveen Werner) (KRBH buffer), and then incubated in KRBH buffer containing 1.67 mM D-glucose at 37 °C for 30 min. Next, islets were washed with KRBH buffer and incubated in KRBH buffer containing 16.7 mM D-glucose for 30 min. Thereafter the islets were washed again and incubated in KRBH buffer containing 40 mM KCl for 30 min. All the incubation media were retrieved at each step, and the islets were collected and homogenized by sonication in 200 µl redistilled water. 50 µl of the sonicates were used for DNA measurement by PicoGreen dsDNA assay kit (Invitrogen), 50 µl of sonicates were mixed with acid-ethanol (0.18 M HCl, 95% ethanol) for insulin extraction at 4 °C overnight. Secreted insulin in the medium and homogenates were measured by mouse insulin Elisa kit (Mercodia, Uppsala, Sweden).

### Lipidomics

Lipid content from 150 pancreatic islets per group were analyzed by ultra-fast liquid chromatography-mass spectrometry (UFLC-MS) based lipidomics analysis and high-performance liquid chromatography by the Swedish Metabolomics Center at the Swedish University of Agricultural Sciences. In detail, the lipid content were extracted following a modified Folch protocol^47–49^. In detail, 200 µL of extraction buffer (2:1 v/v chloroform:methanol) including internal standards (tripalmitin-1,1,1-13C3 and 16:0-d31 ceramide) were added to 150 pancreatic islets. The sample was shaken with a tungsten bead at 30 Hz for 2 minutes in a mixer mill, the beads were removed and 40 µl of 0.1 M NaCl was added. After vortex for 2 min, the samples were let to stand at room temperature for 30 min. The sample was centrifuged at + 4 °C, 14 000 rpm, for 3 minutes. 120 µL of the lower phase were collected and divided into two different microvials (40 + 80 µL) and stored at −80 °C until analysis. The analysis was performed as follows. The set of samples was first analyzed in positive mode. After all samples had been analyzed, the instrument was switched to negative mode and a second injection of each sample was performed. The LC-MS analysis of the lipid extracts were performed as described in Diab *et al*.^47^.

### Histological analysis of pancreas

Pancreata were carefully dissected and fixed in 4% paraformaldehyde (PFA) for 24 h and subsequently processed for paraffin imbedding before 6 µm sections were prepared for immunostaining. Briefly, antigen retrieval was done in target retrieval solution, Citrate pH 6 (Dako, cat no. S2367) while heated at 98 °C for 15 min. Sections were then incubated with primary rat anti-insulin (R&D Systems, MAB1417), rabbit anti-glucagon (Millipore, AB932), goat anti-CD31 (R&D Systems, AF3628) antibodies at 4 °C for 12 h. After primary antibody incubation, sections were incubated with fluorescently labeled secondary antibodies (Invitrogen) at room temperature for 1 h and mounted with ProLong gold antifade mountant with DAPI (Invitrogen).

*In situ* hybridization for *Vegfb* transcripts in pancreas sections were performed using the RNAscope 2.0 FFPE Assay (Advanced Cell Diagnostics) according to the manufacturer’s protocol. RNA Scope® *Vegfb* target probe (Mm-Vegfb-CDS; Advanced Cell Diagnostics) and cell nuclei (DAPI) were visualized with immunofluorescence. Imaging was acquired with a Zeiss LSM 700 confocal microscope and ImageJ software was applied for image analysis as previously described^13^.

### Quantitative RT-PCR

Freshly isolated pancreatic islets and other dissected tissues from around 25 weeks old male mice were snap frozen in liquid nitrogen and kept at −80 °C. Total RNA was isolated with TRIzol reagent (Invitrogen) and Qiagen RNeasy Kit (Qiagen) according to manufacturer’s manual. 1 µg isolated RNA was used for reverse transcription according to manufacturer’s protocol (iScript cDNA synthesis kit, Bio-Rad). qPCR was performed using 25 ng cDNA per reaction using the KAPA SYBR fast qPCR kit (KAPA Biosystems). Expression levels were normalized to mouse ribosomal protein 19 (m*Rpl19*) expression. The following primers were used:

mRpl19: 5′-GGTGACCTGGATGAGAAGGA-3′ and 5′-TTCAGCTTGTGGATGTGCTC-3′

mVegfb: 5′-CCCAGCCACCAGAAGAAA-3′ and 5′-ACATTGCCCATGAGTTCCAT-3′

mIns1: 5′-GGACCTTCAGACCTTGGCGTT-3′ and 5′-GTTGCAGTAGTTCTCCAGCTGGTA-3′

mIns2: 5′-AGCAAGCAGGAAGGTTATTGT-3′ and 5′-GTGTAGAAGAAGCCACGCTC-3′

mGcg: 5′-AGAGGAGAACCCCAGATCATT-3′ and 5′-CGTTTGGCAATGTTGTTCCG-3′

mNrp1: 5′-GGAGCTACTGGGCTGTGAAG-3′ and 5′-CCTCCTGTGAGCTGGAAGTC-3′

mFlt1: 5′-GGAGGAGTACAACACCACGG-3′ and 5′-TTGAGGAGCTTTCACCGAAC-3′

mSlc27a3: 5′-CCTCGGTTTCTCAGGCTCCA-3′ and 5′-CTGTACCGGGCAGGTGTGA-3′

mSlc27a4: 5′-CTATGACTGCCTCCCCCTCT-3′ and 5′-AGTCATGCCGTGGAGTAAGC-3′

mPecam1: 5′-AGAGACGGTCTTGTCGCAGT-3′ and 5′-TACTGGGCTTCGAGAGCATT-3′

mVegfa: 5′-ACTGGACCCTGGCTTTACTG-3′ and 5′-TCTGCTCTCCTTCTGTCGTG-3′

mFlk1: 5′-AGCACCTCTCTCGTGATTTCC-3′ and 5′-AGTAAAAGCAGGGAGTCTGTGG-3′

mCd36: 5′-GAGAACTGTTATGGGGCTAT-3′ and 5′-TTCAACTGGAGAGGCAAAGG-3′

mPlin2: 5′-AGCTCTCCTGTTAGGCGTCTC-3′ and 5′-CGGAGGACACAAGGTCGTAG-3′

### Statistics

Data shown in all figures are presented as mean ± S.E.M. GraphPad Prism software was used. Statistical analyses were performed either by Mann-Whitney test for two groups’ comparison or one-way ANOVA with Dunnett’s multiple comparison test was used for multiple groups’ comparison against *Vegfb*^fl/fl^/RIP-Cre^+/−^ group. A *p* value < 0.05 was considered significant.

## Supplementary information

Supplementary information.
