# Supplementary material for: VEGF-B ablation in pancreatic β-cells upregulates insulin expression without affecting glucose homeostasis or islet lipid uptake
Source: Sci Rep. 2020 Jan 22;10:923. doi: 10.1038/s41598-020-57599-2 (PMC6976647; doi:10.1038/s41598-020-57599-2)
Supplement: Supplementary file 1 — Supplementary information. [file 41598_2020_57599_MOESM1_ESM.pdf]

VEGF-B ablation in pancreatic β-cells upregulate insulin expression without affecting glucose homeostasis or islet lipid uptake

Frank Chenfei Ning[1], Nina Jensen[1], Jiarui Mi[1], William Lindström[1], Mirela Balan[1], Lars Muhl[1], Ulf Eriksson[1], Ingrid Nilsson[1], Daniel Nyqvist[1*]

Supplementary Figure Legends

Supplementary Figure 1

(A) Fasting plasma insulin level of HFD wt, RIP-Cre$^{+/-}$, *Vegfb*$^{fl/fl}$ and *Vegfb*$^{fl/fl}$/RIP-Cre$^{+/-}$ mice (n = 5-12 per group). (B) Comparison of ceramide content and area under curve (AUC) measurement in isolated islets from chow vs. HFD fed wt, RIP-Cre$^{+/-}$, *Vegfb*$^{fl/fl}$ and *Vegfb*$^{fl/fl}$/RIP-Cre$^{+/-}$ mice (n=4-6). Statistics: Mann-Whitney test (B), one-way ANOVA with Dunnett's multiple comparison against *Vegfb*$^{fl/fl}$/RIP-Cre$^{+/-}$ group (A). All data are presented as mean ± S.E.M. *p < 0.05, **p < 0.01, ***p < 0.001, ****p < 0.0001.

Supplementary Figure 2

(A-E) Relative *Nrp1*, *Flt1* (VEGFR-1), *Vegfa*, *Flk1* (VEGFR-2) *and Pecam1* (CD31) mRNA levels in isolated islets from wt, RIP-Cre$^{+/-}$, *Vegfb*$^{fl/fl}$ and *Vegfb*$^{fl/fl}$/RIP-Cre$^{+/-}$ mice (n=5-6 per group). (E-I) Relative *Nrp1*, *Flt1* (VEGFR-1), *Vegfa*, *Flk1* (VEGFR-2) *and Pecam1* (CD31) mRNA levels in isolated islets from HFD wt, RIP-Cre$^{+/-}$, *Vegfb*$^{fl/fl}$ and *Vegfb*$^{fl/fl}$/RIP-Cre$^{+/-}$ mice (n=4-6 per group). Statistics: One-way ANOVA with Dunnett's multiple comparison against *Vegfb*$^{fl/fl}$/RIP-Cre$^{+/-}$ group (A-I). All data are presented as mean ± S.E.M. *p < 0.05.

Supplementary Figure 1

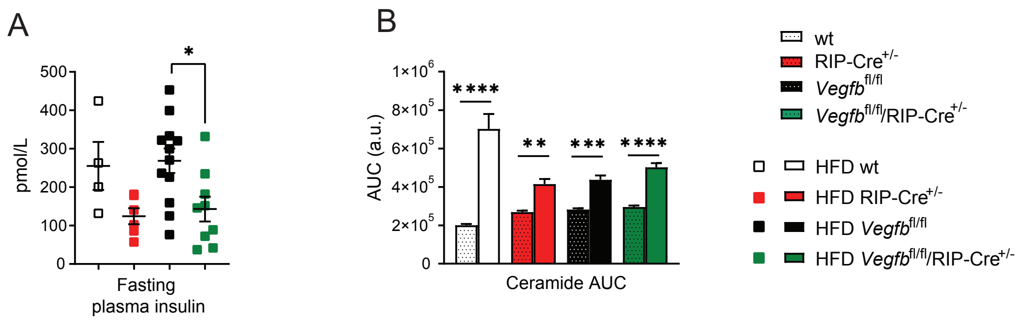

Supplementary Figure 2

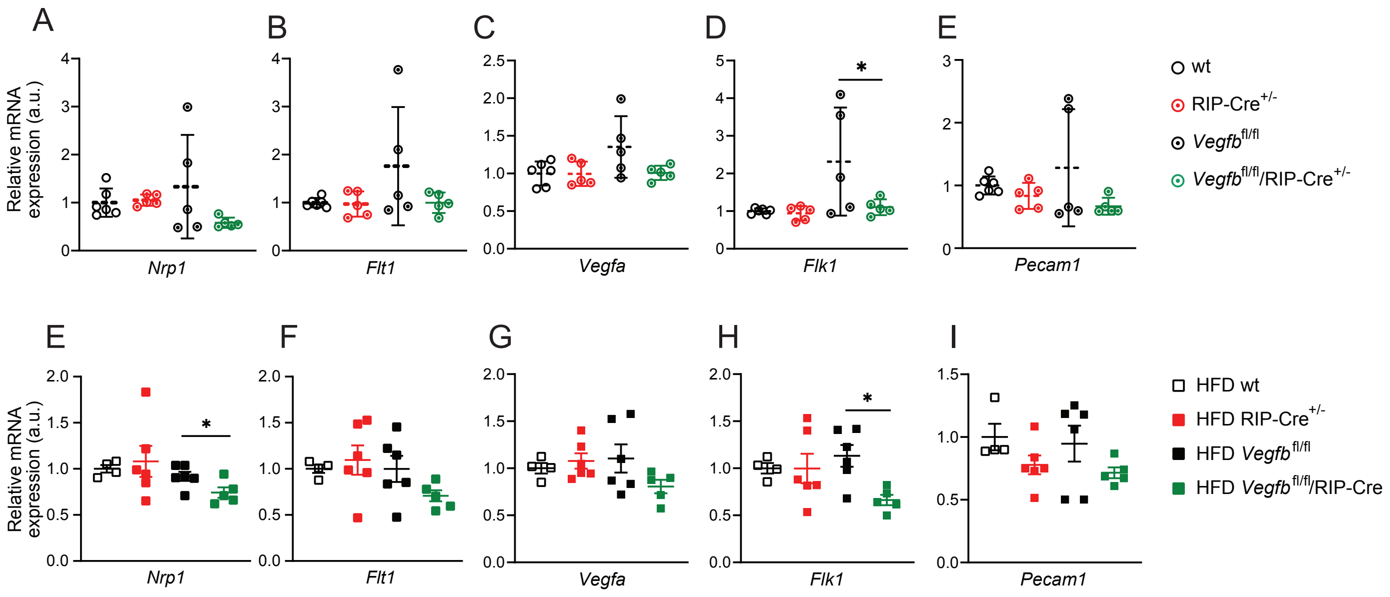

Supplementary Table

Table 1: Real-time quantitative PCR C_T Values from pancreatic islet gene expression analysis

Raw C_T values of real-time quantitative PCR using cDNA from chow and HFD islets. C_T values for housekeeping gene *Rpl19* (L19) are listed separately with gene(s) of interest that has been run on the sample plate. Real-time PCR was performed on Qiagen Rotor-Gene Q machine with 72 wells plates.

Chow Sample	*Rpl19*	*Ins1*	*Vegfb*	*Ins1* ΔCt	*Vegfb* ΔCt
wt 1	15,53	6,72	21,05	8,81	-5,52
wt 2	15,34	7,3	21,08	8,04	-5,74
wt 3	15,21	7,24	21,19	7,97	-5,98
wt 4	15,29	6,75	21,14	8,54	-5,85
wt 5	15,61	6,75	21,29	8,86	-5,68
wt 6	15,4	7,07	21,04	8,33	-5,64
RIP-Cre$^{+/-}$ 1	15,3	6,65	20,79	8,65	-5,49
RIP-Cre$^{+/-}$ 2	15,55	7,34	21,18	8,21	-5,63
RIP-Cre$^{+/-}$ 3	15,39	6,66	21,02	8,73	-5,63
RIP-Cre$^{+/-}$ 4	15,38	7,26	20,85	8,12	-5,47
RIP-Cre$^{+/-}$ 5	15,29	6,71	21,04	8,58	-5,75
Vegfb$^{fl/fl}$ 1	18,19	9,68	23,11	8,51	-4,92
Vegfb$^{fl/fl}$ 2	18,44	9,71	23,38	8,73	-4,94
Vegfb$^{fl/fl}$ 3	15,68	7,63	20,54	8,05	-4,86
Vegfb$^{fl/fl}$ 4	16,07	7,38	21,34	8,69	-5,27
Vegfb$^{fl/fl}$ 5	15,88	7,93	21,15	7,95	-5,27
Vegfb$^{fl/fl}$/RIP-Cre$^{+/-}$ 1	15,97	7,44	23,38	8,53	-7,41
Vegfb$^{fl/fl}$/RIP-Cre$^{+/-}$ 2	15,39	7,27	22,84	8,12	-7,45
Vegfb$^{fl/fl}$/RIP-Cre$^{+/-}$ 3	15,91	7,37	23,61	8,54	-7,7
Vegfb$^{fl/fl}$/RIP-Cre$^{+/-}$ 4	16,42	8,12	23,5	8,3	-7,08
Vegfb$^{fl/fl}$/RIP-Cre$^{+/-}$ 5	15,17	6,44	22,57	8,73	-7,4

Chow Sample	*Rpl19*	*Gcg*	*Ins2*	*Gcg* ΔCt	*Ins2* ΔCt
wt 1	15,23	10,29	6,82	4,94	8,41
wt 2	15,18	9,58	6,92	5,6	8,26
wt 3	15,11	9,15	6,83	5,96	8,28
wt 4	15,2	9,94	6,62	5,26	8,58
wt 5	15,29	10,14	6,74	5,15	8,55
wt 6	15,22	10,08	6,86	5,14	8,36
RIP-Cre$^{+/-}$ 1	15,18	10,06	6,66	5,12	8,52
RIP-Cre$^{+/-}$ 2	15,19	9,92	6,83	5,27	8,36
RIP-Cre$^{+/-}$ 3	15,26	10,46	6,45	4,8	8,81
RIP-Cre$^{+/-}$ 4	15,18	9,22	6,76	5,96	8,42
RIP-Cre$^{+/-}$ 5	14,99	10	6,4	4,99	8,59

Vegfb$^{fl/fl}$ 1	17,93	13,63	8,71	4,3	9,22
Vegfb$^{fl/fl}$ 2	18,24	14,14	10,21	4,1	8,03
Vegfb$^{fl/fl}$ 3	15,37	10,58	6,26	4,79	9,11
Vegfb$^{fl/fl}$ 4	15,74	11,37	6,33	4,37	9,41
Vegfb$^{fl/fl}$ 5	15,76	10,35	6,5	5,41	9,26
Vegfb$^{fl/fl}$/RIP-Cre$^{+/-}$ 1	15,78	11,82	6,35	3,96	9,43
Vegfb$^{fl/fl}$/RIP-Cre$^{+/-}$ 2	15,09	11,1	5,65	3,99	9,44
Vegfb$^{fl/fl}$/RIP-Cre$^{+/-}$ 3	15,69	12,05	6,23	3,64	9,46
Vegfb$^{fl/fl}$/RIP-Cre$^{+/-}$ 4	16,03	11,89	6,64	4,14	9,39
Vegfb$^{fl/fl}$/RIP-Cre$^{+/-}$ 5	14,93	10,91	5,51	4,02	9,42

**Sample**	***Rpl19***	***Nrp1***	***Flt1***	***Nrp1* ΔCt**	***Flt1* ΔCt**
wt 1	15,22	21,47	21,07	-6,25	-5,85
wt 2	15,33	21,46	20,79	-6,13	-5,46
wt 3	15,22	20,9	20,9	-5,68	-5,68
wt 4	15,28	21,32	21,06	-6,04	-5,78
wt 5	15,32	21,66	21,09	-6,34	-5,77
wt 6	15,21	20,52	20,87	-5,31	-5,66
RIP-Cre$^{+/-}$ 1	15,11	21,04	20,89	-5,93	-5,78
RIP-Cre$^{+/-}$ 2	15,4	21,07	20,78	-5,67	-5,38
RIP-Cre$^{+/-}$ 3	15,22	20,91	21,33	-5,69	-6,11
RIP-Cre$^{+/-}$ 4	15,29	21,33	20,68	-6,04	-5,39
RIP-Cre$^{+/-}$ 5	15,06	20,96	21,3	-5,9	-6,24
Vegfb$^{fl/fl}$ 1	17,97	22,86	22,37	-4,89	-4,4
Vegfb$^{fl/fl}$ 2	18,36	22,65	22,31	-4,29	-3,95
Vegfb$^{fl/fl}$ 3	15,42	22,2	21,2	-6,78	-5,78
Vegfb$^{fl/fl}$ 4	15,83	22,71	21,64	-6,88	-5,81
Vegfb$^{fl/fl}$ 5	15,79	21,92	21,28	-6,13	-5,49
Vegfb$^{fl/fl}$/RIP-Cre$^{+/-}$ 1	15,85	22,15	21,26	-6,3	-5,41
Vegfb$^{fl/fl}$/RIP-Cre$^{+/-}$ 2	15,14	22,05	21,39	-6,91	-6,25
Vegfb$^{fl/fl}$/RIP-Cre$^{+/-}$ 3	15,74	22,53	21,21	-6,79	-5,47
Vegfb$^{fl/fl}$/RIP-Cre$^{+/-}$ 4	16,12	22,82	21,84	-6,7	-5,72
Vegfb$^{fl/fl}$/RIP-Cre$^{+/-}$ 5	15,01	21,87	20,8	-6,86	-5,79

**Sample**	***Rpl19***	***Slc27a3***	***Slc27a4***	***Slc27a3* ΔCt**	***Slc27a4* ΔCt**
wt 1	17,26	25,55	23,28	-8,29	-6,02
wt 2	17,27	25,92	23,74	-8,65	-6,47
wt 3	17,21	25,18	23,2	-7,97	-5,99
wt 4	17,3	25,31	23,48	-8,01	-6,18
wt 5	17,4	25,33	23,64	-7,93	-6,24
wt 6	17,29	25,07	22,78	-7,78	-5,49
RIP-Cre$^{+/-}$ 1	17,31	25,06	23,27	-7,75	-5,96
RIP-Cre$^{+/-}$ 2	17,24	25,21	23,16	-7,97	-5,92

RIP-Cre$^{+/-}$ 3	17,31	24,95	23,11	-7,64	-5,8
RIP-Cre$^{+/-}$ 4	17,35	25,26	23,07	-7,91	-5,72
RIP-Cre$^{+/-}$ 5	17,11	24,94	23,13	-7,83	-6,02
Vegfb$^{fl/fl}$ 1	20,01	27,2	25,71	-7,19	-5,7
Vegfb$^{fl/fl}$ 2	20,42	27,27	25,43	-6,85	-5,01
Vegfb$^{fl/fl}$ 3	17,57	26	24,22	-8,43	-6,65
Vegfb$^{fl/fl}$ 4	17,86	26,4	24,93	-8,54	-7,07
Vegfb$^{fl/fl}$ 5	17,82	26,3	24,19	-8,48	-6,37
Vegfb$^{fl/fl}$/RIP-Cre$^{+/-}$ 1	17,9	25,98	24,12	-8,08	-6,22
Vegfb$^{fl/fl}$/RIP-Cre$^{+/-}$ 2	17,23	25,24	23,87	-8,01	-6,64
Vegfb$^{fl/fl}$/RIP-Cre$^{+/-}$ 3	17,24	25,63	24,32	-8,39	-7,08
Vegfb$^{fl/fl}$/RIP-Cre$^{+/-}$ 4	18,13	26,34	24,48	-8,21	-6,35
Vegfb$^{fl/fl}$/RIP-Cre$^{+/-}$ 5	17,01	25,22	23,85	-8,21	-6,84

**Sample**	***Rpl19***	***Vegfa***	***Flk1***	***Vegfa* ΔCt**	***Flk1* ΔCt**
wt 1	15,19	19,05	21	-3,86	-5,81
wt 2	15,23	19,17	20,95	-3,94	-5,72
wt 3	15,19	19,62	20,96	-4,43	-5,77
wt 4	15,29	19,69	21,28	-4,4	-5,99
wt 5	15,59	19,64	21,42	-4,05	-5,83
wt 6	15,36	19,4	21,33	-4,04	-5,97
RIP-Cre$^{+/-}$ 1	15,39	19,23	21,13	-3,84	-5,74
RIP-Cre$^{+/-}$ 2	15,24	19,16	20,9	-3,92	-5,66
RIP-Cre$^{+/-}$ 3	15,27	19,56	21,48	-4,29	-6,21
RIP-Cre$^{+/-}$ 4	15,29	19,63	21,09	-4,34	-5,8
RIP-Cre$^{+/-}$ 5	15,05	19,29	21,4	-4,24	-6,35
Vegfb$^{fl/fl}$ 1	18,03	21,14	22,05	-3,11	-4,02
Vegfb$^{fl/fl}$ 2	18,54	22,28	22,35	-3,74	-3,81
Vegfb$^{fl/fl}$ 3	15,42	19,61	21,12	-4,19	-5,7
Vegfb$^{fl/fl}$ 4	15,86	19,86	21,8	-4	-5,94
Vegfb$^{fl/fl}$ 5	15,91	19,46	20,83	-3,55	-4,92
Vegfb$^{fl/fl}$/RIP-Cre$^{+/-}$ 1	15,9	19,98	21,24	-4,08	-5,34
Vegfb$^{fl/fl}$/RIP-Cre$^{+/-}$ 2	15,28	19,58	21,36	-4,3	-6,08
Vegfb$^{fl/fl}$/RIP-Cre$^{+/-}$ 3	15,81	19,96	21,57	-4,15	-5,76
Vegfb$^{fl/fl}$/RIP-Cre$^{+/-}$ 4	16,19	19,76	21,48	-3,57	-5,29
Vegfb$^{fl/fl}$/RIP-Cre$^{+/-}$ 5	15,08	19,01	20,9	-3,93	-5,82

**Chow Sample**	***Rpl19***	***Pecam1***	***Pecam1* ΔCt**	***L19***	***Plin2***	***Plin2 ΔCt***	***Rpl19***	***Cd36***	***Cd36* ΔCt**
wt 1	15,16	20,56	-5,4	15,32	22,05	-6,73	15,42	21,82	-6,4
wt 2	15,16	20,77	-5,61	15,25	22,13	-6,88	15,25	21,58	-6,33
wt 3	15,11	20,3	-5,19	15,22	22,09	-6,87	15,45	20,86	-5,41
wt 4	15,25	20,68	-5,43	15,28	22,53	-7,25	15,64	22,78	-7,14

wt 5	15,29	20,93	-5,64	15,37	22,7	-7,33	15,72	22,68	-6,96
wt 6	15,22	20,98	-5,76	15,31	22,57	-7,26	15,48	22,5	-7,02
RIP-Cre$^{+/-}$ 1	15,1	20,72	-5,62	15,12	22	-6,88	15,21	22,56	-7,35
RIP-Cre$^{+/-}$ 2	15,25	20,59	-5,34	15,27	22,5	-7,23	15,38	22,55	-7,17
RIP-Cre$^{+/-}$ 3	15,28	21,47	-6,19	15,31	22,96	-7,65	15,42	23,48	-8,06
RIP-Cre$^{+/-}$ 4	15,28	20,95	-5,67	15,33	21,99	-6,66	15,61	21,79	-6,18
RIP-Cre$^{+/-}$ 5	15,04	21,18	-6,14	15,15	22,31	-7,16	15,2	22,55	-7,35
Vegfb$^{fl/fl}$ 1	17,91	22,25	-4,34	17,94	24,73	-6,79	18,09	24,56	-6,47
Vegfb$^{fl/fl}$ 2	18,26	22,5	-4,24	18,55	24,87	-6,32	18,63	25,44	-6,81
Vegfb$^{fl/fl}$ 3	15,38	21,48	-6,1	15,43	23,09	-7,66	15,68	23,77	-8,09
Vegfb$^{fl/fl}$ 4	15,84	22,19	-6,35	15,94	23,485	-7,545	16,03	24,11	-8,08
Vegfb$^{fl/fl}$ 5	15,83	21,25	-5,42	15,95	22,41	-6,46	16,08	22,97	-6,89
Vegfb$^{fl/fl}$/RIP-Cre$^{+/-}$ 1	15,87	21,51	-5,64	15,88	22,95	-7,07	15,96	23,44	-7,48
Vegfb$^{fl/fl}$/RIP-Cre$^{+/-}$ 2	15,19	22,02	-6,83	15,23	23,72	-8,49	15,36	24,56	-9,2
Vegfb$^{fl/fl}$/RIP-Cre$^{+/-}$ 3	15,76	22,02	-6,26	15,82	23,72	-7,9	15,85	24,56	-8,71
Vegfb$^{fl/fl}$/RIP-Cre$^{+/-}$ 4	16,14	22,23	-6,09	16,3	23,24	-6,94	16,37	24,15	-7,78
Vegfb$^{fl/fl}$/RIP-Cre$^{+/-}$ 5	14,97	21,21	-6,24	15,18	22,36	-7,18	15,09	22,74	-7,65

**HFD Sample**	***Rpl19***	***Ins1***	***Vegfb***	***Ins1* ΔCt**	***Vegfb* ΔCt**
wt 1	15,09	6,02	21,41	9,07	-6,32
wt 2	15,07	5,56	20,28	9,51	-5,21
wt 3	15,53	6,25	21,23	9,28	-5,7
wt 4	16	6,61	21,23	9,39	-5,23
RIP-Cre$^{+/-}$ 1	15,06	5,54	20,6	9,52	-5,54
RIP-Cre$^{+/-}$ 2	14,97	5,55	20,44	9,42	-5,47
RIP-Cre$^{+/-}$ 3	14,99	5,39	20,56	9,6	-5,57
RIP-Cre$^{+/-}$ 4	15,31	5,75	21,16	9,56	-5,85
RIP-Cre$^{+/-}$ 5	17,6	8,3	22,85	9,3	-5,25
RIP-Cre$^{+/-}$ 6	17,23	7,87	22,07	9,36	-4,84
Vegfb$^{fl/fl}$ 1	15,76	6,16	21,63	9,6	-5,87
Vegfb$^{fl/fl}$ 2	15,67	6,01	22,04	9,66	-6,37
Vegfb$^{fl/fl}$ 3	15,84	6,46	22,1	9,38	-6,26
Vegfb$^{fl/fl}$ 4	16,89	7,37	22,7	9,52	-5,81
Vegfb$^{fl/fl}$ 5	16,7	6,55	22,66	10,15	-5,96
Vegfb$^{fl/fl}$ 6	16,95	7,2	23,13	9,75	-6,18
Vegfb$^{fl/fl}$/RIP-Cre$^{+/-}$ 1	17,26	8,07	25,1	9,19	-7,84
Vegfb$^{fl/fl}$/RIP-Cre$^{+/-}$ 2	15,38	5,75	23,28	9,63	-7,9
Vegfb$^{fl/fl}$/RIP-Cre$^{+/-}$ 3	15,41	5,5	23,46	9,91	-8,05
Vegfb$^{fl/fl}$/RIP-Cre$^{+/-}$ 4	15,24	5,44	23,34	9,8	-8,1
Vegfb$^{fl/fl}$/RIP-Cre$^{+/-}$ 5	15,39	5,69	23,45	9,7	-8,06

HFD Sample	*Rpl19*	*Gcg*	*Ins2*	*Gcg* ΔCt	*Ins2* ΔCt
wt 1	15,17	11,9	7,1	3,27	8,07
wt 2	15,12	10,28	6,62	4,84	8,5
wt 3	15,68	11,39	7,16	4,29	8,52
wt 4	16,04	11,28	7,89	4,76	8,15
RIP-Cre$^{+/-}$ 1	15,17	10,88	6,66	4,29	8,51
RIP-Cre$^{+/-}$ 2	14,99	10,51	6,58	4,48	8,41
RIP-Cre$^{+/-}$ 3	15,13	10,71	6,52	4,42	8,61
RIP-Cre$^{+/-}$ 4	15,61	11,05	7,12	4,56	8,49
RIP-Cre$^{+/-}$ 5	17,7	14,23	9,16	3,47	8,54
RIP-Cre$^{+/-}$ 6	17,44	13,93	8,85	3,51	8,59
Vegfb$^{fl/fl}$ 1	15,71	12,6	7,15	3,11	8,56
Vegfb$^{fl/fl}$ 2	15,42	12,93	7,11	2,49	8,31
Vegfb$^{fl/fl}$ 3	15,82	11,15	7,39	4,67	8,43
Vegfb$^{fl/fl}$ 4	16,83	12,19	8,33	4,64	8,5
Vegfb$^{fl/fl}$ 5	16,71	14,87	7,32	1,84	9,39
Vegfb$^{fl/fl}$ 6	15,16	12,02	6,51	3,14	8,65
Vegfb$^{fl/fl}$/RIP-Cre$^{+/-}$ 1	17,09	12,77	9,02	4,32	8,07
Vegfb$^{fl/fl}$/RIP-Cre$^{+/-}$ 2	15,14	10,39	7,11	4,75	8,03
Vegfb$^{fl/fl}$/RIP-Cre$^{+/-}$ 3	16,93	15,24	7,72	1,69	9,21
Vegfb$^{fl/fl}$/RIP-Cre$^{+/-}$ 4	15,03	10,86	6,55	4,17	8,48
Vegfb$^{fl/fl}$/RIP-Cre$^{+/-}$ 5	15,12	10,26	7,09	4,86	8,03

HFD Sample	*Rpl19*	*Nrp1*	*Flt1*	*Nrp1* ΔCt	*Flt1* ΔCt
wt 1	15,31	21,23	20,76	-5,92	-5,45
wt 2	15,17	21,13	20,68	-5,96	-5,51
wt 3	15,74	21,83	21,29	-6,09	-5,55
wt 4	16,1	22,28	21,85	-6,18	-5,75
RIP-Cre$^{+/-}$ 1	15,27	21,39	20,64	-6,12	-5,37
RIP-Cre$^{+/-}$ 2	15,2	21,25	20,82	-6,05	-5,62
RIP-Cre$^{+/-}$ 3	15,06	21,33	20,64	-6,27	-5,58
RIP-Cre$^{+/-}$ 4	15,4	22,05	22,05	-6,65	-6,65
RIP-Cre$^{+/-}$ 5	17,74	23,49	22,69	-5,75	-4,95
RIP-Cre$^{+/-}$ 6	17,57	22,73	22,56	-5,16	-4,99
Vegfb$^{fl/fl}$ 1	15,76	21,74	21,12	-5,98	-5,36
Vegfb$^{fl/fl}$ 2	15,55	21,74	21,37	-6,19	-5,82
Vegfb$^{fl/fl}$ 3	15,89	22,42	22,52	-6,53	-6,63
Vegfb$^{fl/fl}$ 4	16,97	23,15	22,76	-6,18	-5,79
Vegfb$^{fl/fl}$ 5	16,43	22,58	21,7	-6,15	-5,27
Vegfb$^{fl/fl}$ 6	16,93	22,91	21,95	-5,98	-5,02

Vegfb$^{fl/fl}$/RIP-Cre$^{+/-}$ 1	17,09	23,21	23,16	-6,12	-6,07
Vegfb$^{fl/fl}$/RIP-Cre$^{+/-}$ 2	15,18	21,81	21,62	-6,63	-6,44
Vegfb$^{fl/fl}$/RIP-Cre$^{+/-}$ 3	15,24	21,77	21,16	-6,53	-5,92
Vegfb$^{fl/fl}$/RIP-Cre$^{+/-}$ 4	15,14	21,53	20,87	-6,39	-5,73
Vegfb$^{fl/fl}$/RIP-Cre$^{+/-}$ 5	15,19	21,91	21,43	-6,72	-6,24

**HFD Sample**	***Rpl19***	***Slc27a3***	***Slc27a4***	***Slc27a3* ΔCt**	***Slc27a4* ΔCt**
wt 1	17,29	25,61	22,82	-8,32	-5,53
wt 2	17,19	25,66	22,93	-8,47	-5,74
wt 3	17,75	26,12	23,15	-8,37	-5,4
wt 4	18,16	27,45	23,68	-9,29	-5,52
RIP-Cre$^{+/-}$ 1	17,19	25,3	22,81	-8,11	-5,62
RIP-Cre$^{+/-}$ 2	17,11	25,19	22,61	-8,08	-5,5
RIP-Cre$^{+/-}$ 3	17,17	25,23	22,85	-8,06	-5,68
RIP-Cre$^{+/-}$ 4	17,45	26,16	23,34	-8,71	-5,89
RIP-Cre$^{+/-}$ 5	19,68	28,62	25,09	-8,94	-5,41
RIP-Cre$^{+/-}$ 6	19,29	27,66	24,32	-8,37	-5,03
Vegfb$^{fl/fl}$ 1	17,76	26,27	23,24	-8,51	-5,48
Vegfb$^{fl/fl}$ 2	17,52	26,15	23,42	-8,63	-5,9
Vegfb$^{fl/fl}$ 3	17,79	27,22	24,29	-9,43	-6,5
Vegfb$^{fl/fl}$ 4	19,24	28,39	25	-9,15	-5,76
Vegfb$^{fl/fl}$ 5	18,5	26,32	23,8	-7,82	-5,3
Vegfb$^{fl/fl}$ 6	19	27,05	24,12	-8,05	-5,12
Vegfb$^{fl/fl}$/RIP-Cre$^{+/-}$ 1	19,07	28,27	25,08	-9,2	-6,01
Vegfb$^{fl/fl}$/RIP-Cre$^{+/-}$ 2	17,14	26,01	23,48	-8,87	-6,34
Vegfb$^{fl/fl}$/RIP-Cre$^{+/-}$ 3	17,24	25,2	22,97	-7,96	-5,73
Vegfb$^{fl/fl}$/RIP-Cre$^{+/-}$ 4	17,02	25,38	22,86	-8,36	-5,84
Vegfb$^{fl/fl}$/RIP-Cre$^{+/-}$ 5	17,15	25,84	23,35	-8,69	-6,2

**HFD Sample**	***Rpl19***	***Vegfa***	***Flk1***	***Vegfa* ΔCt**	***Flk1* ΔCt**
wt 1	15,24	19,31	20,45	-4,07	-5,21
wt 2	15,2	19,01	20,59	-3,81	-5,39
wt 3	15,8	19,63	21,13	-3,83	-5,33
wt 4	16,18	19,85	21,78	-3,67	-5,6
RIP-Cre$^{+/-}$ 1	15,25	18,79	20,81	-3,54	-5,56
RIP-Cre$^{+/-}$ 2	15,15	18,92	20,82	-3,77	-5,67
RIP-Cre$^{+/-}$ 3	15,13	19,05	20,79	-3,92	-5,66
RIP-Cre$^{+/-}$ 4	15,55	19,56	21,83	-4,01	-6,28
RIP-Cre$^{+/-}$ 5	17,75	21,66	22,51	-3,91	-4,76
RIP-Cre$^{+/-}$ 6	17,58	20,93	22,47	-3,35	-4,89
Vegfb$^{fl/fl}$ 1	15,93	19,97	20,8	-4,04	-4,87
Vegfb$^{fl/fl}$ 2	15,69	20	21,09	-4,31	-5,4

Vegfb$^{fl/fl}$ 3	15,92	20,02	21,85	-4,1	-5,93
Vegfb$^{fl/fl}$ 4	17,63	20,86	22,71	-3,23	-5,08
Vegfb$^{fl/fl}$ 5	16,68	19,86	21,96	-3,18	-5,28
Vegfb$^{fl/fl}$ 6	17,02	20,72	21,9	-3,7	-4,88
Vegfb$^{fl/fl}$/RIP-Cre$^{+/-}$ 1	17,14	21,03	22,8	-3,89	-5,66
Vegfb$^{fl/fl}$/RIP-Cre$^{+/-}$ 2	15,24	19,27	21,31	-4,03	-6,07
Vegfb$^{fl/fl}$/RIP-Cre$^{+/-}$ 3	15,27	19,59	21,29	-4,32	-6,02
Vegfb$^{fl/fl}$/RIP-Cre$^{+/-}$ 4	15,1	19,09	20,92	-3,99	-5,82
Vegfb$^{fl/fl}$/RIP-Cre$^{+/-}$ 5	15,24	19,88	21,62	-4,64	-6,38

**HFD Sample**	***Rpl19***	***Vegfa***	***Flk1***	***Vegfa* ΔCt**	***Flk1* ΔCt**
wt 1	15,24	19,31	20,45	-4,07	-5,21
wt 2	15,2	19,01	20,59	-3,81	-5,39
wt 3	15,8	19,63	21,13	-3,83	-5,33
wt 4	16,18	19,85	21,78	-3,67	-5,6
RIP-Cre$^{+/-}$ 1	15,25	18,79	20,81	-3,54	-5,56
RIP-Cre$^{+/-}$ 2	15,15	18,92	20,82	-3,77	-5,67
RIP-Cre$^{+/-}$ 3	15,13	19,05	20,79	-3,92	-5,66
RIP-Cre$^{+/-}$ 4	15,55	19,56	21,83	-4,01	-6,28
RIP-Cre$^{+/-}$ 5	17,75	21,66	22,51	-3,91	-4,76
RIP-Cre$^{+/-}$ 6	17,58	20,93	22,47	-3,35	-4,89
Vegfb$^{fl/fl}$ 1	15,93	19,97	20,8	-4,04	-4,87
Vegfb$^{fl/fl}$ 2	15,69	20	21,09	-4,31	-5,4
Vegfb$^{fl/fl}$ 3	15,92	20,02	21,85	-4,1	-5,93
Vegfb$^{fl/fl}$ 4	17,63	20,86	22,71	-3,23	-5,08
Vegfb$^{fl/fl}$ 5	16,68	19,86	21,96	-3,18	-5,28
Vegfb$^{fl/fl}$ 6	17,02	20,72	21,9	-3,7	-4,88
Vegfb$^{fl/fl}$/RIP-Cre$^{+/-}$ 1	17,14	21,03	22,8	-3,89	-5,66
Vegfb$^{fl/fl}$/RIP-Cre$^{+/-}$ 2	15,24	19,27	21,31	-4,03	-6,07
Vegfb$^{fl/fl}$/RIP-Cre$^{+/-}$ 3	15,27	19,59	21,29	-4,32	-6,02
Vegfb$^{fl/fl}$/RIP-Cre$^{+/-}$ 4	15,1	19,09	20,92	-3,99	-5,82
Vegfb$^{fl/fl}$/RIP-Cre$^{+/-}$ 5	15,24	19,88	21,62	-4,64	-6,38

**HFD Sample**	***Rpl19***	***Pecam1***	***Pecam1* ΔCt**	***Rpl19***	***Plin2***	***Plin2* ΔCt**	***Rpl19***	***Cd36***	***Cd36* ΔCt**
wt 1	15,15	20,32	-5,17	15,25	22,14	-6,89	15,3	21,45	-6,15
wt 2	15,18	20,9	-5,72	15,24	21,93	-6,69	15,25	21,55	-6,3
wt 3	15,68	21,42	-5,74	15,76	22,37	-6,61	15,85	22,39	-6,54
wt 4	16,02	21,74	-5,72	16,1	22,73	-6,63	16,19	22,46	-6,27
RIP-Cre$^{+/-}$ 1	15,18	20,98	-5,8	15,21	22,23	-7,02	15,29	22,37	-7,08

RIP-Cre$^{+/-}$ 2	15	20,82	-5,82	15,09	22,07	-6,98	15,23	22,1	-6,87
RIP-Cre$^{+/-}$ 3	14,98	20,96	-5,98	15,07	22,08	-7,01	15,2	22,23	-7,03
RIP-Cre$^{+/-}$ 4	15,36	21,88	-6,52	15,69	22,84	-7,15	15,76	22,83	-7,07
RIP-Cre$^{+/-}$ 5	17,69	23,14	-5,45	17,74	24,73	-6,99	17,82	25,34	-7,52
RIP-Cre$^{+/-}$ 6	17,2	23,22	-6,02	17,58	24,57	-6,99	18,02	25,71	-7,69
Vegfb$^{fl/fl}$ 1	15,71	20,91	-5,2	15,79	22,33	-6,54	15,78	21,54	-5,76
Vegfb$^{fl/fl}$ 2	15,57	20,9	-5,33	15,73	22,28	-6,55	15,64	20,81	-5,17
Vegfb$^{fl/fl}$ 3	15,86	22,42	-6,56	15,83	23,19	-7,36	15,93	22,85	-6,92
Vegfb$^{fl/fl}$ 4	17,85	23,09	-5,24	18,15	24,24	-6,09	18,51	25,44	-6,93
Vegfb$^{fl/fl}$ 5	16,62	21,94	-5,32	16,62	23,22	-6,6	16,7	22,03	-5,33
Vegfb$^{fl/fl}$ 6	16,99	22,47	-5,48	17,06	24,05	-6,99	17,11	23,49	-6,38
Vegfb$^{fl/fl}$/RIP-Cre$^{+/-}$ 1	17,06	23,06	-6	17,06	24,04	-6,98	17,17	24,24	-7,07
Vegfb$^{fl/fl}$/RIP-Cre$^{+/-}$ 2	15,27	21,55	-6,28	15,19	22,5	-7,31	15,34	22,53	-7,19
Vegfb$^{fl/fl}$/RIP-Cre$^{+/-}$ 3	15,22	20,99	-5,77	15,3	22,25	-6,95	15,25	22,4	-7,15
Vegfb$^{fl/fl}$/RIP-Cre$^{+/-}$ 4	15,12	21,26	-6,14	15,1	22,29	-7,19	15,15	22,41	-7,26
Vegfb$^{fl/fl}$/RIP-Cre$^{+/-}$ 5	15,25	21,36	-6,11	15,29	22,47	-7,18	15,33	21,56	-6,23
